# Report from the 31st Meeting on Toxinology, “Toxins: Playing with and Fighting Them!”, Organized by the French Society for Toxinology on 1–2 December 2025

**DOI:** 10.3390/toxins18030138

**Published:** 2026-03-11

**Authors:** Sylvie Diochot, Raphaële Le Garrec, Michel M. Dugon, Pascale Marchot

**Affiliations:** 1 Institut de Pharmacologie Moléculaire et Cellulaire (IPMC), Université Côte d’Azur, CNRS, INSERM, Sophia Antipolis, 06560 Valbonne, France; 2 Laboratoire Interactions Epithéliums Neurones (LIEN), Université de Bretagne Occidentale, 29200 Brest, France; rlegarrec@univ-brest.fr; 3 Venom Systems Lab, Ryan Institute, School of Natural Sciences, University of Galway, H91TK33 Galway, Ireland; michel.dugon@universityofgalway.ie; 4 Architecture et Fonction des Macromolécules Biologiques (AFMB) Laboratory, Faculté des Sciences, Campus Luminy, CNRS/Aix-Marseille Université, Case 932, 13288 Marseille CEDEX 09, France

**Keywords:** algal toxin, animal toxin, bacterial toxin, fungal toxin, plant toxin, toxin evolution, intoxination, mode of action, neuropharmacology, serotherapy, venom biodiversity, venomics

## Abstract

The French Society for Toxinology (SFET) held its 31st annual meeting (RT31) on 1–2 December 2025 at Hôtel Le Saint Paul in Nice, France, on the famous French Riviera. The meeting, which gathered 75 participants from around the world, was organised there for the second consecutive year, while previous editions were all held in Paris. The RT31 main theme, “Toxins: Playing with and fighting them”, explored recent, cutting-edge research in the field of animal venoms and of toxins from algal, animal, bacterial, fungal, plant and microbial origins, in emphasizing the evolution of the toxins, their modes of action and roles, and ways of counteracting intoxinations. These key topics were largely covered through 26 oral and 18 poster communications, organized into three main thematic areas covering three specific aspects of toxinology, along with a traditional fourth, more general session enabling participants to present recent data outside of these themes but nevertheless providing valuable information to the field. This report presents the abstracts of nine of the invited lectures, 14 of the selected lectures, and 16 of the posters, in accordance with the authors’ agreement to publish them. Also, we announce the winners of the “Best Oral Communication” and “Best Poster Communication” awards, which recognize the outstanding contributions of young researchers and their inventive work in toxinology.

## 1. Preface

**Figure 1:**
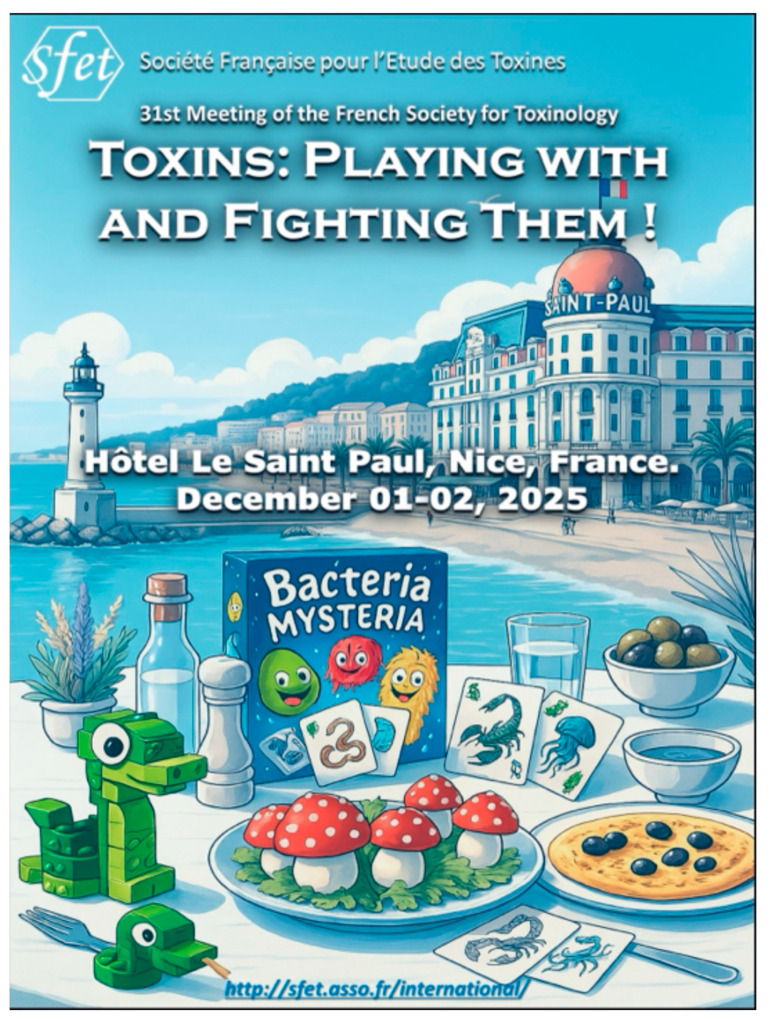
The poster announcing the 31st Meeting on Toxinology (RT31) of the French Society for Toxinology (SFET).

The 31st Meeting on Toxinology (RT31) of the French Society for Toxinology (SFET) (http://sfet.asso.fr/international, English site accessed on 16 February 2010) was held on 1–2 December 2025 at Hôtel Le Saint Paul in Nice, France. After 29 editions in Paris, that was the second time that the meeting was held on the French Riviera. The central theme of the year was “Toxins: Playing with and fighting them”, as reflected in the announcement poster (Figure 1).

**Figure 2:**
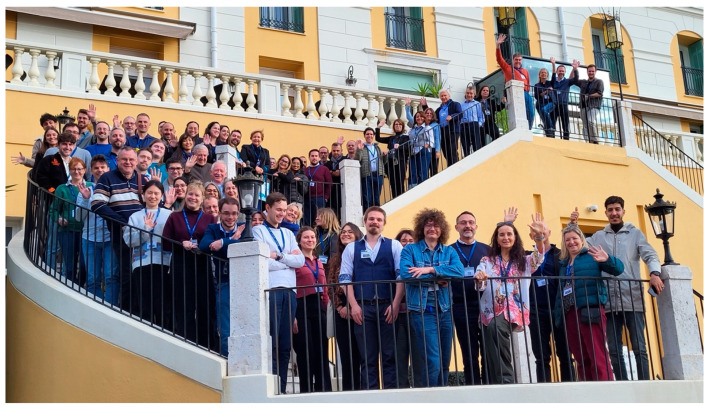
Most—but not all—of the participants at the 31st Meeting on Toxinology (RT31) of the French Society for Toxinology (SFET).

The RT31 meeting welcomed a total of 75 participants, with women accounting for 45% and men for 55% of attendees (Figure 2). Among them, 21 participants came from 11 European countries other than France, including Belgium, the Czech Republic, Denmark, England, Germany, Ireland, Italy, Portugal, Slovenia, Spain, and Sweden, while 22 participants came from other regions of the world, including Algeria, Australia, Brazil, Ecuador, French Guiana, Lebanon, Morocco, Tunisia, and the United States of America. Overall, participants from abroad represented 54% of the total attendance, a ratio highlighting the increasing appeal of the SFET meetings within the international toxinology community. This worldwide audience comprised Master and Ph.D. students (23%), early-career and established researchers (72%), and industrial company members (5%), reflecting a wide range of expertise and career levels. Participants from diverse geographical and scientific backgrounds gathered to exchange knowledge, strengthen existing collaborations, establish new partnerships and discuss recent advances in toxinology. The strong international representation underlines once again the growing global interest in the scientific topics discussed at the meeting. In this context, SFET meetings continue to be an essential forum for promoting scientific progress and fostering international cooperation in the fields of venomology and toxinology.

The central theme of the year, “Toxins: Playing with and fighting them”, was well developed through 26 invited and selected lectures. These presentations highlighted the extreme diversity of toxins of animal, plant, or bacterial origin, their dynamic evolution over time (traced using biotechnological tools such as multi-omics), and their respective impact on the physiological functions of living organisms. Some presentations explored the complexity of venoms, which contain a multitude of toxins, many of which are still unknown or unexploited and could provide new pharmacological avenues for biomedical research. Data from genomics, pharmacology, immunology, screening, proteomics, and other approaches aimed at understanding the functions of toxins were reported in the various presentations. Three main themes were documented: “Evolution of toxins” (six presentations), “Intoxination and countermeasures” (six presentations), “Modes of action and roles of toxins” (seven presentations), along with a “miscellaneous” theme allowing six people to present other topics in venomology and toxinology.

The 10 experts in toxinology invited to the RT31 meeting came from a wide range of backgrounds (Italy, Portugal, Germany, Tunisia, Denmark, Sweden, England, France) and presented (i) biotechnological developments enabling a better approach to and understanding of the wide diversity of toxins in the animal, plant, and bacterial worlds, (ii) highly specific molecular tools enabling immunological diagnosis and optimization of treatments for envenomation or intoxication, and (iii) the molecular aspect of the mode of action of toxins such as snake venom metalloproteinases, bacteria, and a plant toxin acting on ion channels. The newly included plenary lecture provided an overview of the use of toxins in neuropharmacology and their future in therapeutics.

The 16 other speakers, selected from among researchers, post-doctoral fellows and students, presented topics relating to the various themes chosen. Two presentations reported on the discovery of new toxins from ant venoms, with an in-depth analysis of their behaviour, evolution, and diversity, demonstrating the antimicrobial and immunomodulatory power of these toxins. Others focused on public health with rapidly evolving serotherapy tools. Still others highlighted the mode of action and potential benefits of bacterial or animal toxins for the treatment of autoimmune diseases. Finally, various presentations addressed original topics such as the richness of wasp venoms, the presence of pathogens in scorpion venoms, cases of envenomation during pregnancy, and the adaptation of ant-predatory spider venoms.

A total of 18 posters were presented by researchers, post-doctoral fellows and students during this RT31 meeting, of which six, presented by young participants, were selected for poster highlights. The poster presentations explored how natural toxins interact with living systems and how they can be both harmful and medically useful. Together, they examined how these molecules move through the environment, affect tissues, immune responses, and nerve or vascular functions, and how their impacts can be visualized using advanced analytical tools. Several studies focused on the discovery of new bioactive compounds and the evaluation of their ability to inhibit pathological processes such as inflammation, abnormal cell growth, or ageing. Others developed innovative methods for detecting, measuring, or neutralizing toxins. Overall, the RT31 main theme linked environmental chemistry, biology, and biotechnology to better understand and harness animal, plant, phycotoxins and bacterial toxins.

**Figure 3:**
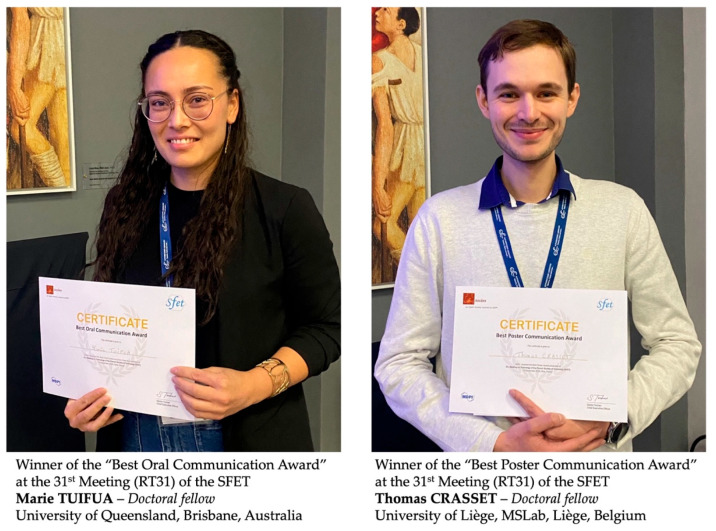
The winners of the “Best Oral Communication Award” and “Best Poster Communication Award” granted by Toxins (MDPI) for the 31st Meeting on Toxinology (RT31) of the French Society for Toxinology (SFET).

Thanks to the generous support of the journal *Toxins* (MDPI), two prizes of 250 € each were awarded to young toxinologists: one for the best oral communication and the other for the best poster communication (Figure 3). The winners were selected by a jury composed of invited speakers and members of the SFET Board of Directors present at the RT31 meeting and representing a wide diversity of expertise in toxinology.

## 2. Scientific and Organizing Committee (SFET Board of Directors in 2025)

The RT31 Organizing Committee was composed of the 12 members of the 2025 Board of Directors, who are all scientific researchers specializing in venomology and toxinology.

- Elsa Bonnafé, Biochimie et Toxicologie des Substances Bioactives (BTSB), INU Champollion, Albi, France.
- Katrina Campbell, Institute for Global Food Security, Queen’s University Belfast, Belfast, Northern Ireland.
- Sylvie Diochot, Institut de Pharmacologie Moléculaire et Cellulaire (IPMC), Valbonne, France.
- Michel Dugon, Galway Venom System Lab, The Ryan Institute, Galway, Ireland
- Sébastien Dutertre, Institut des Biomolécules Max Mousseron (IBMM), Montpellier, France.
- Daniel Ladant, Institut Pasteur, Paris, France.
- Raphaële Le Garrec, Université Bretagne Occidentale, laboratoire Interactions Epithéliums-Neurones (LIEN), Brest, France.
- Christian Legros, Université d’Angers, Angers, France.
- Emmanuel Lemichez, Unité des Toxines Bactériennes, Institut Pasteur, Paris, France
- Pascale Marchot, Centre National de la Recherche Scientifique (CNRS)/Aix-Marseille Université, Marseille, France.
- Loïc Quinton, Université de Liège, Liège, Belgium.
- Michel Ronjat, retired from l’Institut du Thorax, Université de Nantes, Nantes, France.

## 3. Invited Lectures

Ten experts in toxinology were formally invited to present their work as 30 min lectures; the abstracts of nine of them are presented below (when more than one author, the underlined name is that of the presenter).

### 3.1. Marine Animal Forests: An Unexplored Source of New Venom Toxins

Maria Vittoria Modica ^1,^*, Serena Leone ^2^, Dany Dominguez-Perez ^3^, Alberto Pallavicini ^4^, Marco Gerdol ^4^ and Sébastien Dutertre ^5^

^1^  Department of Biology and Evolution of Marine Organisms, Stazione Zoologica Anton Dohrn, Roma, Italy
^2^  Department of Biology and Evolution of Marine Organisms, Stazione Zoologica Anton Dohrn, Napoli, Italy
^3^  Department of Biology and Evolution of Marine Organisms, Stazione Zoologica Anton Dohrn, Amendolara, Italy
^4^  Department of Life Sciences, University of Trieste, Italy
^5^  Institut des Biomolecules Max Mousseron, CNRS UMR 5247, Montpellier, France
*  Correspondence: mariavittoria.modica@szn.it

**Abstract:** Marine animal forests are peculiar benthic ecosystems occurring worldwide from the tropics to the poles and from the shallows to the deep sea, and are dominated by tropical and cold-water coral reefs, black coral and gorgonian gardens. Research on marine venoms has been focusing on sea anemones, while other Cnidaria lineages have been largely understudied. Cnidaria from temperate, mesophotic and cold-water marine animal forests are mostly heterotrophic, relying on venom to prey on zooplankton, which makes them particularly appealing for toxin characterization. We have recently investigated venom composition in several species of *Octocorallia* and *Hexacorallia* with a broad bathymetric range collected from the upper and lower mesophotic areas. Using transcriptomics and proteomic data, we identified multiple potential venom neurotoxins and cytotoxins, some of which belong to established venom protein families (e.g., PLA2, ShK, Kunitz, actinoporins), while others display novel folds and are worth further functional characterization. We used available genomic data to reconstruct the evolutionary history of *Octocorallia*-specific toxin families. This study broadens the range of known anthozoan toxins, highlighting the relevance of expanding biodiscovery to novel habitats and targets.

**Keywords:** Anthozoa; proteomics; transcriptomics

### 3.2. Evolutionary Diversification of Animal Toxins: Multiomics Elucidation and Biotechnological Relevance

Agostinho Antunes ^1,2,^*

^1^  CIIMAR/CIMAR, Interdisciplinary Centre of Marine and Environmental Research, University of Porto, Terminal de Cruzeiros do Porto de Leixões, Av. General Norton de Matos, Porto 4450–208, Portugal
^2^  Department of Biology, Faculty of Sciences, University of Porto, Rua do Campo Alegre, Porto 4169-007, Portugal
*  Correspondence: aantunes@ciimar.up.pt

**Abstract:** Animal toxins play critical roles in ecological processes such as predation, defence, and interspecies competition. Due to their remarkable molecular diversity, the evolutionary dynamics of many of these bioactive compounds remain poorly understood. Integrating multi-layered omics approaches can uncover vast reservoirs of previously uncharacterized molecules, far beyond what is catalogued in existing toxin databases. In parallel, machine learning has emerged as a powerful tool for identifying the biomedical potential of these compounds, particularly functional peptides with diverse therapeutic activities, including antimicrobial, antifungal, antiviral, and anticancer properties. Cross-species omics analyses of animal toxins thus represent a promising avenue for discovering novel pharmacologically active peptides, offering significant potential for therapeutic innovation and translational research.

**Keywords:** animal toxin; functional peptide; omics; venom

### 3.3. Functional Venomics-Guided Insights into the Evolution and Chemical Diversity of Spider Venoms

Tim Lüddecke ^1,2,3,^*

^1^  Fraunhofer Institute for Molecular Biology and Applied Ecology, Ohlebergsweg 12, 35392 Gießen, Germany
^2^  LOEWE-Centre for Translational Biodiversity Genomics, Senckenberganlage 25, 60325 Frankfurt am Main, Germany
^3^  Institute for Insect Biotechnology, Justus Liebig University of Gießen, Heinrich-Buff-Ring 28-32, 35392 Gießen, Germany
*  Correspondence: tim.lueddecke@ime.fraunhofer.de

**Abstract:** Spiders constitute one of the most taxonomically and ecologically diverse clades of terrestrial venomous organisms. Their venoms, characterized by a complex assemblage of short, cysteine-rich peptides with high specificity for neuronal targets, have garnered substantial interest within pharmaceutical research as potential sources of novel therapeutics. Despite the proliferation of translational studies exploring the pharmacological potential of isolated venom components, our understanding of the evolutionary trajectories and ecological roles of spider venoms remains remarkably limited. Moreover, the vast majority of global spider biodiversity remains unsampled with respect to venom composition, rendering spider venoms a largely unexplored frontier—aptly described as “toxinological dark matter”. Recent advances in systems biology, comparative genomics, and biotechnological platforms have begun to illuminate this molecular landscape, enabling more integrative analyses of venom evolution, function, and diversification. In this presentation, I will highlight emerging frameworks and key discoveries that contribute to a more cohesive understanding of spider venom biology, emphasizing evolutionary context and functional innovation.

**Keywords:** arachnid; evolution; venomics

### 3.4. Cobra Venoms: Mode of Action and Neutralizing Performance of Highly Specific Nanobodies

Balkiss Bouhaouala-Zahar ^1,2,^*, Hiba Mejri ^1,3^, Rym Mokrani ^4^, Ayoub Ksouri ^1^, Mabrouk Seddik ^5^, Nour Awad ^6^, Gabriel Ayme ^7^, Thouraya Chagour ^1^, Ahlem Mokrani ^4^, Charraf Eddine Louchene ^4^, Imed Salhi ^5^, Rahma Benabderrazek ^1^, Rym Benkhalifa ^1^, Zakaria Benlasfar ^1^, Akbar Oghalaie ^7^, Delavar Shabazzadeh ^7^, Pierre-Jean Corringer ^6^, Mohamed Hammadi ^5^, Selma Djilani ^4^ and Pierre Lafaye ^3^

^1^  Laboratory of Venoms and Theranostic Applications (LR20IPT01), Place Pasteur, BP704, Pasteur Institute of Tunis, Université Tunis el Manar, Tunis 1002, Tunisia
^2^  Medical School of Tunis, Université Tunis el Manar, Tunis 1002, Tunisia
^3^  Antibody Engineering Platform, C2RT, Université de Paris Cité, CNRS UMR 3528, Institut Pasteur, 75015 Paris, France
^4^  Research and Development Laboratory, Institut Pasteur Algérie, University of Algiers 1, Algiers 16000, Algeria
^5^  Livestock and Wildlife Laboratory (LR16IRA04), Arid Lands Institute (I.R.A), University of Gabès, Medenine 4119, Tunisia
^6^  Channel Receptors Unit, Université de Paris Cité, CNRS UMR 3571, Institut Pasteur, 75015 Paris, France
^7^  Venom and Biotherapeutics Molecules Laboratory, Medical Biotechnology Department, Biotechnology Research Center, Pasteur Institute of Iran, Tehran, Iran
*  Correspondence: balkiss.bouhaouala@fmt.utm.tn

**Abstract:** Snakebite envenoming (SBE) remains a severely neglected public health issue, particularly affecting tropical and subtropical regions, leading to high morbidity and mortality rates, especially across Africa and Asia. Of particular concern are *Naja* sp. cobra venoms with highly diffusing neuro- and neuromuscular toxins responsible for fatal respiratory paralysis. Given the significant risks associated with *Naja haje* SBE, there are important disease-correlated challenges that need to be addressed. The COBRA NGaV project seeks to deliver novel nanobody-based antisera to tackle cobra bite envenoming. This study represents the first investigations of *Naja* sp. in the MENA context. The dual strategy consists of the characterization of venom toxic fractions from cobra specimens captured for the first time in Algeria, Iran and Tunisia biotopes and in vitro assays to evaluate their interactions with acetylcholine receptors, coupled with the use of an innovative nanobody-based next-generation of SBE antisera. To this aim, healthy dromedaries received the less toxic venom fractions as the first two doses, thus minimizing potential harm to the animal. Upon adjusted immunization protocols and dromedary healthcare monitoring, libraries were constructed from cDNA encoding VHH IgG domains isolated from peripheral lymphocytes. The toxin-specific nanobodies were selected via phage-display library screening and selection options. We demonstrated that the combination of the best nanobody binders is required to achieve improvement in neutralizing capacities and reaching 100% neutralizing efficacy of lethal venom fraction doses in mice, demonstrating the synergism of cobra toxin effects. Altogether our findings indicate the potential of the developed nanobodies to serve as a novel antivenom therapy. Presently, we intensified our dual strategy by proteomic and genomic approaches to get an outstanding molecular understanding of toxin mode of action and subsequently better cobra toxin fighting.

**Keywords:** cobra venom; LD50 lethal dose; *Naja haje*; nanobody; neutralizing capacity; toxin

### 3.5. Next-Generation Therapeutics for Snakebite Envenoming: De Novo Design from Bench to Bite

Timothy P. Jenkins *

Digital Biotechnology Lab., Technical University of Denmark, Denmark

*  Correspondence: tpaje@dtu.dk

**Abstract:** The discovery of toxin-binding nanobodies has opened new possibilities for understanding and counteracting the molecular mechanisms of snake venom toxins. In a recent publication, we reported an integrated pipeline that combines high-throughput selection to isolate nanobodies capable of neutralizing complex snake toxin targets. While this approach produced highly potent neutralisers, it also revealed the practical limitations of current discovery workflows, which remain labour-intensive and time-consuming. To overcome these bottlenecks, our group has explored the complementary use of de novo protein design. Earlier this year, we demonstrated that generative design pipelines can create functional binders entirely from scratch within only a few weeks. Using AI-guided protein design and sequence optimization, we achieved in vivo neutralization of several toxin targets, showing that design can now rival and surpass traditional discovery in both quality and speed. Our current work extends this paradigm further by focusing on what we term “massive polyspecificity”—the rational design of binders that can recognize entire toxin families while maintaining strong and selective interactions. Through smart, sophisticated design approaches, we aim to capture shared motifs across related toxins to generate broadly neutralizing molecules with predictable behaviour. Together, these efforts illustrate a shift from discovery-driven to design-driven therapeutic development. By merging generative computational design with high-throughput experimental data, we envision a future where new antitoxins can be conceived, optimized, and validated in record time. This convergence of discovery and design not only accelerates research but also opens new routes for creating better therapeutics at unprecedented speed.

**Keywords:** de novo design; nanobody; toxin

### 3.6. From Emerging Pathogens to Biotechnology: Functional and Structural Insights into Novel Bacterial Toxins

Geoffrey Masuyer ^1,2,^*

^1^  Department of Biochemistry and Biophysics, Stockholm University, 10691 Stockholm, Sweden
^2^  Department of Life Sciences, Centre for Therapeutic Innovation, University of Bath, BA2 7AY, Bath, UK
*  Correspondence: geoffrey.masuyer@dbb.su.se

**Abstract:** Following the recent identification of numerous new members of the botulinum-like toxin family, efforts were made to identify additional bacterial toxins that could pose a threat to human health or offer potential biotechnological applications. Mono-ADP-ribosyl transferase (mART) proteins are secreted virulence factors produced by several human pathogens and include the diphtheria toxin and *Pseudomonas aeruginosa* exotoxin A (PE). A PE-like toxin was recently identified in *Aeromonas hydrophila* and implicated in human cases of necrotising fasciitis. By mining genomic databases, six additional putative PE homologues were discovered, significantly expanding this toxin family across a diverse range of bacterial species. Comparative genomic analyses suggest that the broad distribution of PE-like toxins is driven by horizontal gene transfer, underscoring the importance of ongoing surveillance of emerging pathogens and their virulence factors. Structural and functional characterization of these newly identified PE-like toxins revealed conserved features that may be exploited for the development of novel toxin-based therapeutics, including immunotoxins and targeted drug delivery platforms.

**Keywords:** ADP-ribosyl transferase; bacterial toxin; biotechnology; emerging pathogen

### 3.7. Production and Characterization of Cytotoxic and Haemotoxic Snake Venom Metalloproteinases

Sophie Hall ^1^, Iara Aimê Cardoso ^1^, Mark C. Wilkinson ^2^, Maria Molina Carretero ^3^, Srikanth Lingappa ^1^, Bronwyn Rand ^1^, Dakang Shen ^1^, Johara Boldrini-França ^1^, Richard Stenner ^1^, Georgia Balchin ^1^, Konrad Hus ^1^, Renaud Vincentelli ^4^, Andrew Mumford ^3^, Nicholas R. Casewell ^2^, Imre Berger ^1^ and Christiane Schaffitzel ^1,^*

^1^  School of Biochemistry, University of Bristol, University Walk, Bristol BS8 1TD, UK.
^2^  Centre for Snakebite Research and Interventions, Liverpool School of Tropical Medicine, Liverpool L3 5QA, UK
^3^  School of Cellular and Molecular Medicine, University of Bristol, 1 Tankard’s Close, Bristol BS8 1TD, UK
^4^  Architecture et Fonction des Macromolécules Biologiques (AFMB) laboratory, Faculté des Sciences–Campus Luminy case 932, 163 avenue de Luminy, 13288 Marseille CEDEX 09, France
*  Correspondence: christiane.berger-schaffitzel@bristol.ac.uk

**Abstract:** Snake venoms contain variable mixtures of toxins that evolved to incapacitate prey but cause extensive pathology in snakebite patients. In viper venom, the most potent toxins are the haemorrhagic and coagulopathic snake venom metalloproteinases (SVMPs). SVMP research has been hampered by the lack of a generic recombinant production protocol. Using a baculovirus/insect cell expression protocol, we produced and functionally validated enzymes from all three structurally variable SVMP classes (PI, PII and PIII). Incorporating the native N-terminal prodomain, which blocks the active site via a cysteine switch motif, overcame the cytotoxicity of SVMPs. Incubation with Zn^2+^ activated the SVMP zymogens, resulting in degradation of the PIII prodomain. Functional validation of the recombinant SVMPs was performed using substrate degradation, platelet aggregation and blood coagulation assays, in comparison with a native venom-purified SVMP. Our study provides a platform for the future expression of SVMPs of value for bioprospecting and discovery of novel snakebite therapeutics.

**Keywords:** cytotoxicity; haemotoxicity; snake venom metalloproteinase; zymogen

### 3.8. Exploring Epigenetic Reprogramming by Legionella pneumophila During Infection

Monica Rolando *

Biologie des Bactéries Intracellulaires, Département de Microbiologie, Institut Pasteur, Paris, France

* Correspondence: mrolando@pasteur.fr

**Abstract:** Understanding how pathogens manipulate the epigenetic regulation of the host to proliferate and survive may help find new strategies to fight infectious diseases. One of these pathogens is *Legionella pneumophila*, a bacterium that replicates naturally in aquatic amoeba, but can also infect human cells and cause a severe pneumonia in humans, called Legionnaires’ disease. Uniquely, *L. pneumophila* encodes a large repertoire of proteins containing eukaryotic-like motifs acquired from its hosts and translocated via a specialized T4SS called Dot/Icm. Some of these proteins target the host cell nucleus and reprogram the cellular response to the bacterial advantage. We have shown that some of these effectors, called nucleomodulins, directly modify the host chromatin by altering post translational modifications of histone proteins. Additionally, we recently identified a secreted effector that reshapes the nuclear functions by targeting non-histone proteins, predominantly RNA binding proteins that play multiple regulatory roles in the nucleus, such as subnuclear body formation, transcriptional regulation, genome stability and alternative splicing. Studying the molecular mimicry employed by *L. pneumophila* not only provides new insights into how this bacterium hijacks the host cell response to cause Legionnaires’ disease, but also helps in identifying new regulatory mechanisms of the host.

**Keywords:** epigenetics; host–pathogen interaction; nucleomodulin; T4SS effector

### 3.9. Resiniferatoxin, a Tool to Mitigate Heat Increases—An In Vitro Approach

César Mattei *

Laboratoire MitoVasc, UMR CNRS 6015—INSERM 1083, bâtiment IRIS-2, 3 rue Roger Amsler, 49100 Angers, France

*  Correspondence: cesar.mattei@univ-angers.fr

**Abstract:** Exertional heatstroke is a condition characterized by an acute crisis in which the patient’s temperature rises above 40 °C, which can have severe acute effects that can lead to coma and possibly death. There is currently no specific treatment, and management begins by immersing the individual in a cold-water bath. This condition may be contextual or due to a genetic defect in intracellular calcium signalling. Various mutations in calcium channels are known to cause hyperthermic conditions. Several channelopathies have been described based on variants of the TRPV1 channel. It is known that TRPV1 channel agonists—capsaicin or resiniferatoxin (RTX)—induce a significant decrease in body temperature. At the cellular level, heat production occurs in the mitochondria, with temperatures approaching 50 °C. We have therefore identified and characterized a human mitochondrial variant of TRPV1 (named mitoTRPV1) using a multidisciplinary approach. By expressing it in HEK cells, we show that this channel is specifically present in the inner mitochondrial membrane and dissipates calcium currents. The pharmacological activation of mitoTRPV1 with RTX induces mitochondrial cooling without modification of mitochondrial respiration and ATP production. With a genetic variant of mitoTRPV1 from an individual prone to heatstroke, there is a total loss-of-function, including the absence of thermal regulation. We conclude that mitoTRPV1 acts as a cellular thermostat to promote mitochondrial thermolysis without impairing respiration and ATP production. These data point to the potential implication of mitoTRPV1 in human diseases related to temperature dysregulation and suggest that specifically targeting mitoTRPV1 could enable the treatment of hyperthermia disorders.

**Keywords:** heat dissipation; heatstroke; mitochondria; resiniferatoxin; TRPV1

## 4. Oral Presentations

Sixteen confirmed and young toxinologists were selected on abstracts to present their work as 20 min lectures; the abstracts of 15 of them are presented below (when more than one author, the underlined name is that of the presenter).

### 4.1. Composition and Role of Foaming Venom in Ants

Léonie Rosay ^1,^*, Samuel Robinson ^2^, Nathan Tene ^3^, Michel Treihlou ^3^, Elsa Bonnafé ^3^, Arnaud Billet ^3^, Jérôme Orivel ^4^ and Axel Touchard ^5^

^1^  Université de Guyane, UMR EcoFoG, Campus Agronomique, BP 316, Kourou CEDEX 97379, France
^2^  Institute for Molecular Bioscience, The University of Queensland, Queensland, Australia
^3^  BTSB, Université de Champollion, Place de Verdun, Albi 81012, France
^4^  CRBE (Université de Toulouse/CNRS/IRD/Toulouse INP), 118 route de Narbonne, 31062 Toulouse CEDEX, France
^5^  CNRS, UMR EcoFoG, Campus Agronomique, BP 316, Kourou CEDEX 97379, France
*  Correspondence: leonie.rosay@yahoo.com

**Abstract:** Ants have evolved diverse venom usage strategies that allow them to adapt to a wide range of ecological contexts. One of the most remarkable strategies is the ability of certain ponerine ants from two non-sister genera (*Pachycondyla* and *Pseudoneoponera*) to produce and release foaming venom via their stings. However, the mechanisms underlying foam production and its ecological role are not well understood. To address this gap, we conducted a comparative proteo-transcriptomic analysis of venom composition across six species of *Pachycondyla* and *Pseudoneoponera*. Some *Pachycondyla* species produce foam, while others do not. This provides an opportunity to identify proteins or peptides that are convergently present in foaming venoms but absent in non-foaming ones. In parallel, behavioural assays were conducted to examine the functional role of foaming venom in interactions with other ants and termites to identify the ecological contexts that trigger its release. Our preliminary findings on three species reveal distinct interspecific differences in venom peptide and protein repertoires but also several peptides with notable sequence similarities across foaming species. Further characterization will allow broader comparisons and may identify the molecule(s) responsible for foam production. Additionally, behavioural assays suggest that foaming venom primarily serves a defensive function, providing mechanical protection against smaller enemies/competitors, such as ants and termites.

**Keywords:** ant; foam; peptide; proteo-transcriptomic; venom

### 4.2. Investigating Pain-Causing Toxins in Plant Venoms

Marie Tuifua ^1,^ *, Jennifer Deuis ^1^, Irina Vetter ^1,2^, Thomas Durek ^1^ and Samuel Robinson ^1^

^1^  Institute for Molecular Biosciences, The University of Queensland, Brisbane, QLD 4072
^2^  School of Pharmacy, The University of Queensland, Brisbane, QLD 4102
*  Correspondence: uqmtuifu@uq.edu.au

**Abstract:** Stinging plants are widespread globally, with certain species posing health risks to humans. Notably, *Dendrocnide* species in Australia and *Urtica ferox* in New Zealand are known for their painful stings. While extensive research has been conducted on their morphology, the composition and full effects of their venom remain poorly understood.

Recent studies have identified nociceptive peptides, gympietides from *Dendrocnide* species [1] and urticatoxins from *U. ferox* [2], which activate voltage-gated sodium channels, suggesting a common molecular mechanism underlying their pain-inducing effects observed in vivo.

Although *Urtica* and *Dendrocnide* plants appear to share a similar defensive strategy, our findings reveal that their venom compositions are distinct. Further investigation into the mode of action of *Dendrocnide* venom suggests a unique mechanism involving lipid transfer proteins (LTPs) and gympietides. While LTPs are commonly found across the plant kingdom, this is the first time they have been identified as components of a plant venom, highlighting a novel functional role for these molecules in plant defence.

This research project aims to elucidate the evolutionary relationship between *Dendrocnide* and *Urtica*, to investigate the role of LTPs as a venom component, and ultimately to better understand plant venom mechanisms of action in human pain pathways.

**Keywords:** pain; peptide toxin; plant venom

**References:**

1. Gilding, E.K.; Jami, S.; Deuis, J.R.; Israel, M.R.; Harvey, P.J.; Poth, A.G.; Rehm, F.B.H.; Stow, J.L.; Robinson, S.D.; Yap, K.; et al. Neurotoxic peptides from the venom of the giant Australian stinging tree. *Sci. Adv.* **2020**, *6*, eabb8828.
2. Xie, J.; Robinson, S.D.; Gilding, E.K.; Jami, S.; Deuis, J.R.; Rehm, F.B.H.; Yap, K.; Ragnarsson, L.; Chan, L.Y.; Hamilton, B.R.; et al. Neurotoxic and cytotoxic peptides underlie the painful stings of the tree nettle *Urtica ferox*. *J. Biol. Chem.* **2022**, *298*, 102218.

### 4.3. Discovery of a Human Monoclonal scFv Antibody Against Moojase, a Snake-Derived Serine Protease

Marcela Romanazzi ^1^, Eloise Trostdorf Monteiro Filardi ^1^, Geovanna Maria Malachias-Pires ^1^, Jomanah Alqallaf ^2^, Jarred Williams ^2^, José Rafael De Almeida ^2^, Felipe Augusto Cerni ^3^, Sakthivel Vaiyapuri ^2^ and Manuela B. Pucca ^1,4,^ *

^1^  Graduate Program in Bioscience and Biotechnology Applied to Pharmacy, School of Pharmaceutical Sciences, São Paulo State University, Araraquara 19060-900, Brazil
^2^  School of Pharmacy, University of Reading, Reading RG6 6UB, UK
^3^  Medical School, Federal University of Roraima, Boa Vista 69310-000, Brazil
^4^  Department of Clinical Analysis, School of Pharmaceutical Sciences, São Paulo State University (UNESP), Araraquara 19060-900, Brazil
*  Correspondence: manuela.pucca@unesp.br

**Abstract:** Snakebite envenoming remains a major neglected tropical disease, and antivenoms currently available often present significant limitations, including low specificity, batch-to-batch variability, and potential adverse reactions. Monoclonal antibodies (mAbs) represent a promising alternative due to their high specificity, reproducibility, and safety. This study aimed to discover, produce, purify, and characterise human monoclonal antibodies against moojase, a serine protease derived from *Bothrops moojeni* venom, using a phage display strategy with the Griffin I antibody fragment library. Through multiple rounds of biopanning against purified moojase, several antibody candidates were isolated. Two clones exhibited particularly strong binding profiles during the selection process and were subsequently expressed and purified for detailed characterisation. Preliminary affinity measurements by biolayer interferometry (BLI) revealed that these antibodies bound more strongly to moojase than a commercially available antivenom, which was used as a positive control. Moreover, initial cross-reactivity assays suggested that at least one selected antibody could also recognise other serine proteases or additional venom components, as demonstrated by enhanced binding to crude venom preparations. Ongoing in vitro studies are further evaluating the neutralising potential and functional activity of these antibodies. These include coagulation profiling using rotational thromboelastometry (ROTEM) as well as enzymatic activity assays designed to assess their ability to inhibit the moojase proteolytic function. In addition, these assays will be extended to examine cross-neutralization potential against venoms from other *Bothrops* species, thereby providing insight into the breadth of antibody activity and their applicability in the development of broad-spectrum antivenoms. Collectively, these findings support the feasibility of using phage display-derived human monoclonal antibodies as a novel strategy for developing next-generation antivenoms with enhanced specificity, potency, and cross-species efficacy against *Bothrops* envenoming.

**Keywords:** antivenom; BLI; *Bothrops*; moojase; phage display; scFv

### 4.4. Snakebite Roraima: Addressing a Neglected Health Issue in Brazil’s Most Overlooked State

Felipe Augusto Cerni ^1,^ * and Manuela B. Pucca ^2^

^1^  Medical School, Federal Univ. of Roraima, Boa Vista, Brazil
^2^  Department of Clinical Analysis, School of Pharmaceutical Sciences, São Paulo State University, Araraquara, São Paulo, Brazil
*  Correspondence: felipe_cerni@hotmail.com

**Abstract:** Snakebite envenoming has long remained an underestimated public health issue, despite the global occurrence of approximately 2.7 million cases and nearly 130,000 deaths annually. Because snakebite predominantly affects impoverished populations, the absence of research funding has resulted in a corresponding lack of innovation. In Brazil, snakebite is officially recognized as a major neglected tropical disease, with over 28,000 cases recorded annually, mostly in the Amazon region. Roraima, the northernmost Brazilian state, is situated entirely within the Amazon rainforest and sustains the highest incidence of snakebite in the country, reaching 99.5 cases per 100,000 inhabitants (by comparison, about 45 cases per 100,000 are recorded in the neighboring state of Amazonas). However, the actual number of accidents in Roraima may be underreported because of limited research investments, the presence of a large indigenous population, and the recent influx of Venezuelan migrants. Additionally, Roraima has the weakest economy in Brazil and a fragile health system, marked by shortages of physicians and essential medical supplies. The Snakebite Roraima research group investigates diverse aspects of envenoming, including studies in basic and clinical toxinology, alongside a recently established Educational Snakebite Program (@snakebiteroraima). This presentation will address the neglected reality of snakebite in Roraima, covering epidemiology, pathophysiology of envenoming, barriers to treatment, and the resulting physical and social consequences. Furthermore, an overview of the educational initiative designed to mitigate these challenges and reduce snakebite impact in the state will be provided, aligned with the objectives of the Snakebite Global Initiative 2017. The Snakebite Roraima Educational Program receives support from the Hamish Ogston Foundation.

**Keywords:** Amazonia; Roraima; snakebite

### 4.5. Viper Venom–Derived Lebecetin as a Modulator of Av-Integrins in Neuroinflammatory and Demyelinating Disorders

Nour-Elhouda Neili ^1^, Zaineb Abdelkafi-Koubaa ^1,2^, Jed Jebali ^1^, Ghada Sahraoui ^2,3^, Melika Ben Ahmed ^3,4^, Najet Srairi-Abid ^1^, Naziha Marrakchi ^1^, Raoudha Doghri ^2,3^ and Ines ElBini ^5,^*

^1^  Experimental Platform of Neurosciences and Neurodegeneration (EPNN), Laboratory of Biomolecules, Venoms and Theranostic Applications (LR20IPT01), Pasteur Institute of Tunis, University of Tunis, El Manar, Tunis, Tunisia
^2^  Research Laboratory of Precision Medicine/Personalized Medicine and Oncology Investigation (LR21SP01), Saleh Azaiez Institute, Tunis, Tunisia
^3^  Faculty of Medicine of Tunis, University of Tunis, El Manar, Tunis, Tunisia
^4^  Laboratory of Transmission, Control and Immunobiology of Infections (LR16IPT02), Pasteur Institute of Tunis, University of Tunis, El Manar, Tunis, Tunisia
*  Correspondence: ines.bini@pasteur.tn

**Abstract:** Neurodegenerative disorders such as multiple sclerosis and Parkinson’s disease are characterized by alterations in myelin content and structure, processes in which transmembrane receptors like integrins may play a critical role. This study examined the involvement of αv-integrins in experimental models of neuroinflammation and demyelination using lebecetin, a C-type lectin purified from *Macrovipera lebetina* venom, as an αv-integrin modulator. In a neuroinflammatory model, lebecetin suppressed the upregulation of αv, β3, β5, α5, and β1 integrins, reduced the release of the pro-inflammatory cytokine IL-6 and chemokine CXCL-10, and lowered expression of phosphorylated NF-κB. In indirect astrocyte–oligodendrocyte co-cultures, lebecetin counteracted the downregulation of αv/β3 and upregulation of β1 associated with decreased levels of the myelin basic protein (MBP). Direct treatment of oligodendrocytes with lebecetin restored both integrin and MBP expression. Western blot analyses further revealed that lebecetin enhanced PI3K and phosphorylated mTOR expression while reducing phosphorylated AKT, indicating that its neuroprotective and pro-myelinating effects may involve the PI3K/mTOR/AKT intracellular signaling pathway. In vivo, lebecetin promoted remyelination, evidenced by increased MBP expression in the brains of cuprizone-treated mice. Collectively, these findings implicate αv-integrins in both neuroinflammation and demyelination and highlight αv-integrin targeting as a promising therapeutic strategy for demyelinating diseases [1].

**Keywords:** demyelination; integrin; neuroinflammation; oligodendrocyte; snake venom

**References:**

1. Neili, N.E.; AbdelKafi-Koubaa, Z.; Jebali, J.; Kaidi, K.; Sahraoui, G.; Ahmed, M.B.; Srairi-Abid, N.; Marrakchi, N.; Doghri, R.; ElBini, I. Modulation of αv integrins by lebecetin, a viper venom-derived molecule, in experimental neuroinflammation and demyelination models. *Sci. Rep.* **2024**, *14*, 22398.

### 4.6. Clostridium perfringens Epsilon Toxin and Multiple Sclerosis

Michel R. Popoff ^1,^* and Marie-Lise Gougeon ^2^

^1^  18T rue Brignole Galliera, 92140 Clamart, France
^2^  Unité Immunité Innée et Virus, Institut Pasteur, 25 rue du Dr Roux, 75724 Paris CEDEX 015
*  Correspondence: popoff2m@gmail.com

**Abstract:** Multiple sclerosis (MS) is a chronic immune-mediated neurological disorder, characterized by progressive demyelination and neuronal cell loss in the central nervous system. Many possible causes of MS have been proposed, including genetic factors, environmental triggers, and infectious agents. Recently, *Clostridium perfringens* epsilon toxin (ETX) has been incriminated in MS, based initially on the isolation of the bacteria from one MS patient, combined with an immunoreactivity to ETX [1]. Moreover, two surveys showed a more prevalent immunoreactivity to ETX in people with MS than in healthy controls [2].

ETX-producing *C. perfringens* is responsible for an acute and rapidly fatal disease in sheep, goats, and more rarely cattle, termed enterotoxemia. ETX produced in the intestine of animals passes the intestinal barrier, disseminates through the blood circulation, crosses the blood brain barrier, and targets specific cells in the central nervous system such as granule cells, oligodendrocytes, and brain endothelial vascular cells. A hallmark of ETX biological activity is ETX targeting oligodendrocytes leading to demyelination as shown in mice and rats as well as cell models [3]. ETX induces multifocal and inflammatory demyelination in experimental mice which is consistent with lesions observed in MS patients [2,3]. However, no ETX production has been evidenced in MS patients [2].

To investigate a putative causative role of ETX in MS, we analysed the pattern of antibodies reacting to the toxin using a sensitive quantitative immunoassay. This prospective observational study included one hundred patients with relapsing remitting multiple sclerosis (RRMS), all untreated, and ninety matched healthy controls. By assessing the isotypic pattern and serum concentration of ETX-reacting antibodies, our study shows a predominant IgM response over IgG and IgA antibody responses both in MS patients and controls, and significantly higher levels of IgM reacting to ETX in MS patients compared to the control group. A longitudinal follow-up of ETX-specific antibody response in a subgroup of MS patients did not show any correlation with disease evolution [4]. Overall, these unexpected findings are not compatible with a specific recognition of ETX by serum antibodies from MS patients. They rather argue for a cross-immunological reactivity with an antigen, possibly an autoantigen, mimicking ETX [4].

**Keywords:** *Clostridium perfringens*; epsilon toxin; multiple sclerosis

**References:**

1. Rumah, K.R.; Linden, J.; Fischetti, V.A.; Vartanian, T. Isolation of *Clostridium perfringens* type B in an individual at first clinical presentation of multiple sclerosis provides clues for environmental triggers of the disease. *PLoS ONE* **2013**, *8*, e76359.
2. Ma, Y.; Sannino, D.; Linden, J.R.; Haigh, S.; Zhao, B.; Grigg, J.B.; Zumbo, P.; Dündar, F.; Butler, D.; Profaci, C.P.; et al. Epsilon toxin-producing *Clostridium perfringens* colonize the multiple sclerosis gut microbiome overcoming CNS immune privilege. *J. Clin. Investig.* **2023**, *133*, e163239.
3. Popoff, M.R. Epsilon toxin: A fascinating pore-forming toxin. *FEBS J.* **2011**, *278*, 4602–4615.
4. Gougeon, M.L.; Seffer, V.; Hoxha, C.; Maillart, E.; Popoff, M.R. Does *Clostridium perfringens* epsilon toxin mimic an auto-antigen involved in multiple sclerosis? *Toxins* **2025**, *17*, 27.

### 4.7. The Arsenal of Brazilian Scorpion Toxins: Venom Diversity, Bioprospecting, and Novel Insights into NETosis

Manuela B. Pucca *

Faculty of Pharmaceutical Sciences, São Paulo State University, Araraquara, SP, Brazil

*  Correspondence: manuela.pucca@unesp.br

**Abstract:** Brazilian scorpion venoms represent a complex arsenal of bioactive molecules, of which many remain poorly characterized. The study of these venoms is not only essential to understand the pathophysiology of envenomation but it also offers promising opportunities for the discovery of novel therapeutic and biotechnological tools. Our investigations have demonstrated that Brazilian scorpion venoms (from the Southeast to the North), although distinct in their protein profiles, share a remarkable abundance of neurotoxins that act on ion channels. These molecules are key mediators of the clinical manifestations of scorpion stings, such as intense pain, autonomic disturbances, and cardiovascular complications. Importantly, our group was among the first to show that scorpion neurotoxins are capable of inducing neutrophil extracellular trap formation (NETosis), linking envenomation to innate immune responses and inflammation in a pioneering way. This finding expands the understanding of scorpion toxin activity beyond neurotoxicity and highlights their relevance as modulators of host defense mechanisms. Despite the progress achieved, many Brazilian scorpion venoms remain underexplored. Comprehensive proteomic and functional analyses are still lacking for several species, especially those from the Amazon region. Our recent fieldwork has led to the description of a new scorpion species and even a new genus from northern Amazonia, reinforcing how much biodiversity remains hidden and how crucial it is to investigate these venoms before their potential is lost. The bioprospecting of Brazilian scorpion toxins thus represents a dual opportunity: to deepen knowledge of the molecular and physiological mechanisms underlying envenomation and to identify molecules with potential biomedical applications, ranging from ion channel modulators to candidates for anti-inflammatory or antimicrobial therapies.

**Keywords:** biodiversity; NETosis; neurotoxin

### 4.8. Investigating the Role of the ExoY Effector in Pseudomonas aeruginosa Virulence

Vincent Deruelle ^1,^*, Gabrielle Dupuis ^1,2^, Dorothée Raoux-Barbot ^1^, Jose Roberto Ponce Lopez ^1^, Daniel Ladant ^1^ and Undine Mechold ^1^

^1^  Institut Pasteur, Unité de Biochimie des Interactions Macromoléculaires, Département de Biologie Structurale et Chimie, CNRS UMR 3528, Paris, France
^2^  Centre de Recherche Saint-Antoine, Mucoviscidose: Physiopathologie et Phénogénomique, UMRS 938, Paris, France
*  Correspondence: vincent.deruelle@pasteur.fr

**Abstract:** *Pseudomonas aeruginosa* is a Gram-negative opportunistic pathogen frequently implicated in hospital-acquired infections. Using its Type 3 Secretion System (T3SS), the bacterium injects four effectors: ExoS, ExoT, ExoU, and ExoY, into host cells to manipulate cellular functions and exacerbate disease. While ExoS, ExoT, and ExoU are known to directly promote cytotoxicity, the contribution of ExoY remains ambiguous despite its widespread presence among clinical and environmental isolates. ExoY is a nucleotidyl cyclase that hijacks host cyclic nucleotide signaling by generating supraphysiological levels of cGMP, and, to a lesser extent, cAMP, cUMP and cCMP. Given that ExoY is co-injected with the other T3SS effectors, we explored whether ExoY activity modulates the cytotoxic effects of ExoS and ExoT. Substitutions were therefore performed in ExoY nucleotide binding pocket to alter its substrate specificity, favoring cAMP production over cGMP. These ExoY variants were validated in vitro using enzymatic assays and with stable luminescent reporter lines. Then, recombinant *P. aeruginosa* strains expressing ExoT or ExoS with these modified ExoY effectors were used to infect various host cell lines and cytotoxicity was real-time monitored. Our findings revealed two major insights. First, intracellular ExoY-catalyzed cGMP production modulates T3SS-dependent cytotoxicity and secondly, in turn, T3SS toxins can suppress ExoY activity according to the cellular context. These findings highlight the intricate interplay between T3SS effectors and uncover a novel layer of complexity based on ExoY in the pathogenic strategy of *P. aeruginosa*.

**Keywords:** interplay; *P. aeruginosa*; T3SS effector

### 4.9. Integrative Peptidomics and Bioinformatic Analysis of Four Wasp Venoms Reveals Diverse Peptides and Novel Cryptides

Kai Wang ^1,2^, Jiangtao Qiao ^1,2^, Gauthier Eppe ^3^, Loïc Quinton ^3^, Eric Haubruge ^2,^* and Hongcheng Zhang ^1,4^

^1^  State Key Laboratory of Resource Insects, Institute of Apicultural Research, Chinese Academy of Agricultural Sciences, Beijing, China
^2^  Terra Research Center, Gembloux Agro-Bio Tech, University of Liege, Gembloux, Belgium
^3^  Laboratory of Mass Spectrometry, MolSys Research Unit, University of Liege, Liege, Belgium
^4^  Key Laboratory of Bee Products for Quality and Safety Control, Ministry of Agriculture and Rural Affairs, Beijing, China
*  Correspondence: e.haubruge@uliege.be

**Abstract:** Bee venom therapy has been officially integrated into traditional Chinese medicine and is widely used as an adjunct treatment for various diseases. In comparison, wasp venom, with its complex composition and potent biological activities, remains a valuable yet underexplored source of bioactive molecules. Systematic investigation of wasp venom peptides and their pharmacological potential is therefore essential for new drug discovery and the rational utilization of venom-derived resources. In this study, venoms from four wasp species (*Vespa mandarinia*, *V. velutina*, *V. basalis*, and *Provespa barthelemyi*) were collected by electrical stimulation and analyzed using liquid chromatography–tandem mass spectrometry (LC–MS/MS). A total of 681 peptides were identified, nearly 90% of which represent novel sequences. Comparative analysis revealed that most peptides were species-specific, while only a few were shared among species. These results indicate notable differences and an apparent species-dependent pattern in wasp venom peptidomes. Further analysis suggested that peptide release in wasp venom may be regulated by specific proteolytic cleavage patterns, which could contribute to structural and functional diversity. Bioinformatic predictions indicated that 291 peptides possess potential pharmacological activities, including angiotensin-converting enzyme (ACE) and dipeptidyl peptidase IV (DPP4) inhibition, suggesting possible therapeutic applications in hypertension and diabetes. In addition, ten novel cryptides were identified, highlighting potential for venom-derived drug discovery. Overall, this study provides the first comprehensive, side-by-side analysis of the peptidome from four wasp venoms. It characterizes their peptide diversity, generation mechanisms, and predicted bioactivities, offering valuable insights for future pharmaceutical and biotechnological development.

**Keywords:** bioactivity; bioinformatic analysis; drug discovery; novel cryptide; peptidomics; wasp venom

### 4.10. Prevalence and Interaction of Microbes Within the Venom System of Scorpions

Dayle Leonard ^1,2,^*, Aoife Boyd ^2^ and Michel M. Dugon ^1^

^1^  Venom Systems lab, Ryan Institute, School of Natural Sciences, University of Galway, H91TK33, Galway, Ireland
^2^  Pathogenic Mechanisms Research Group, School of Natural Sciences, University of Galway, H91TK33, Galway, Ireland
*  Correspondence: d.leonard21@universityofgalway.ie

**Abstract:** Over 1.2 million scorpion stings are recorded globally every year, resulting in over 3000 deaths. The long-term effects of scorpion envenoming have not been systematically studied, although organ defects and severe infections, which may further lead to disfigurement, amputations and occasionally death, have been reported in the literature. These infections are often masked by venom-led symptoms or are thought to be part of the envenoming syndrome, leading to delays in treatment. We investigated the presence of microbial communities associated with the venom system of the Moroccan Brown scorpion *Scorpio mogadorensis*, as well as the potential presence of antimicrobial agents in the venom of a selection of scorpion species from North Africa, Latin America and West Asia. Specimens and soil samples were collected from three habitats with varying degree of synanthropic disturbances in the Tiout oasis, located on the foothill of the Anti-Atlas Mountains of Southern Morocco. Microbial communities were isolated from the metasoma, venom gland and aculeus tissue samples, and identified using culture-dependent and culture-independent methods. Comparative bioinformatic analysis was conducted to identify microbial communities unique to the scorpion venom system, including potential pathogens, and highlight links between scorpion and soil microbiomes. In addition, we assessed the venom of seventeen species of scorpion for antimicrobial and anti-biofilm activity using minimum inhibitory concentration and crystal violet assays. Overall, 112 bacterial isolates including *Staphylococcus aureus* and seven fungal isolates were obtained. Culture independent methods revealed over 6000 amplicon sequence variants including *Escherichia* spp. Results showed that (i) potential pathogens of medical importance are present on and within the venom system of scorpions and may be “flushed” into the victim during a stinging event; (ii) scorpions produce anti-biofilm compounds as part of their venom, potentially as a defence against infectious microbes; (iii) the venom of the two North African buthids, *Androctonus crassicauda* and *A. mauritanicus*, inhibit biofilm formation by methicillin-resistant and methicillin-sensitive *S. aureus* by 80–90% and by *E. coli* by 60–70%.

**Keywords:** anti-biofilm; microbiome; scorpion

### 4.11. Scorpionism and Pregnancy: Assessing Teratogenicity and Placental Cell Responses to Tityus serrulatus Venom

Isabela Caroline Dos Santos ^1,^*, Marcos William Gualque ^1,2^, Ana Marisa Fusco Almeida ^1,2^, José Martin Murrieta-Coxca ^3,4^, Udo R. Markert ^3,4^ and Manuela B. Pucca ^1,2^

^1^  Graduate Program in Bioscience and Biotechnology Applied to Pharmacy, School of Pharmaceutical Sciences, São Paulo State University (UNESP), Campus Araraquara, 19060-900, Brazil
^2^  Department of Clinical Analysis, School of Pharmaceutical Sciences, São Paulo State University, Araraquara 19060-900, Brazil
^3^  Placenta Lab. Department of Obstetrics, Jena University Hospital Jena, Germany
^4^  Center for Early Pregnancy and Reproductive Health (CEPRE; www.uniklinikum-jena.de/cepre, accessed on 16 February 2020), funded by the German Federal Ministry of Research, Technology and Space (BMFTR; funding reference: 01GR2305A)
*  Correspondence: isabela.caroline@unesp.br

**Abstract:** Envenomations by venomous animals remain a major global public health challenge. In Brazil, the scorpion *Tityus serrulatus* is considered the most dangerous species and the one with the widest distribution across the country, accounting for approximately 200,000 reported stings annually. Its potent venom contains low molecular weight proteins, known as neurotoxins, which act on voltage-gated sodium and potassium channels, leading to excessive neurotransmitter release and triggering a pro-inflammatory “cytokine storm.” Evidence suggests that the venom is associated with foetal malformations, intrauterine death, and spontaneous abortions, largely due to its ability to stimulate uterine contractions. Despite advances in understanding the mechanisms of scorpion envenomation, the pathological effects of the venom in pregnant women and its ability to cross the placental barrier remain poorly understood. This study aims to evaluate the teratogenic potential of *T. serrulatus* venom using the zebrafish (*Danio rerio*) alternative model and to analyze the response of human placental cells (BeWo and JEG-3). In teratogenicity assays, embryos were exposed to varying concentrations of venom and evaluated over 72 h post-fertilization. Effects were assessed based on developmental abnormalities such as coagulation, somite malformation, delayed development, tail deformation, and absence of heartbeat (OECD 236). For assays with human placental cells, trophoblastic BeWo and JEG-3 cells were used to measure changes in ATP production as an indicator of metabolic activity. In teratogenicity tests, several malformations were observed under experimental conditions. At the highest concentration (100 µg/mL), the survival rate of zebrafish embryos at 72 h post-fertilization was approximately 30%. In addition, experiments with cellular models showed that ATP production levels indicated significant alterations in cellular metabolic activity caused by the venom. Thus, we conclude that the scorpion venom demonstrated teratogenic potential under the tested conditions, with atypical morphologies suggesting that the venom was able to cross the chorion of the embryos and induce malformations. The venom increases metabolic activity (ATP production); these metabolic changes may be related to the venom effects but require further investigation. As next steps, we intend to investigate, using more advanced systems such as placental perfusion and the placenta-on-a-chip model, whether the neurotoxins present in the venom can cross the placental barrier. This study will contribute to understanding the still poorly known effects of scorpion venom, opening new perspectives for research in reproductive toxicology with advanced placental models.

**Keywords:** human placental model; scorpion venom; zebrafish

### 4.12. Affinity Capture of Toxin Ligands Using Mass Spectrometry

Lou Freuville ^1,2,^*, Rudy Fourmy ^3^, Aude Violette ^3^, Alain Brans ^4^ and Loïc Quinton ^1^

^1^  Mass Spectrometry Laboratory, MolSys Research Unit, Department of Chemistry, University of Liège, Allée du Six Août, 11—Quartier Agora, 4000, Liège, Belgium
^2^  Laboratory of Molecular Cancer Biology, URPhyM, NARILIS, Faculty of Medicine, University of Namur, rue de Bruxelles, 51, 5000, Namur, Belgium
^3^  Alphabiotoxine Laboratory sprl, Montroeul-au-bois, 7911, Belgium
^4^  Centre for Protein Engineering, University of Liège, B-4000 Liège, Belgium
*  Correspondence: lfreuville@uliege.be

**Abstract:** Animal venoms are complex natural mixtures containing many biologically active peptides, exhibiting high selectivity and potency against (among other targets) membrane receptors, such as G-protein coupled receptors (GPCRs). These venom-derived toxins provide valuable tools for exploring receptor function and have significant potential in pharmacophore modelling for drug discovery. Despite the growing interest in venoms as a source of therapeutic leads, the field remains underdeveloped, largely due to the complexity of venom components and the low-throughput nature of existing screening techniques targeting multiple molecular receptors of interest.

To identify new venom-derived peptide toxins, we designed an innovative methodology that combines affinity capture on cell membranes with mass spectrometry. The approach was first validated using the human vasopressin type 2 receptor (hV2R) and mambaquaretin-1, a well-characterized GPCR ligand from green mamba venom. The method was then extended to nicotinic acetylcholine receptors (nAChRs), by selectively fishing α-neurotoxins directly from crude and fractionated *Naja kaouthia* venom.

Practically, the membranes of cells overexpressing receptors were incubated with venom HPLC fractions. Toxins displaying affinity for the receptor bound to the membranes, while those without affinity remained in solution. Bound fractions were analysed by MALDI-MS and LC-MS/MS proteomics.

Our results validate the effectiveness of this affinity-based “ligand-fishing” strategy coupled with proteomics, which enabled the direct discovery of membrane receptor-targeting toxins from venom fractions, and even from crude venom. They also establish a proof of concept for higher-throughput venom screening, opening new opportunities to explore peptide candidates across a wide range of molecular targets.

**Keywords:** affinity capture; ligand fishing; mass spectrometry

### 4.13. Efficacy of Euryphagous Spider Venom Against Ants

Andrea Quattrocolo, Ondrej Michalek and Stano Pekár *

Masaryk University, Terrestrial Invertebrate Research Group, Brno, Czech Republic

*  Correspondence: pekar@sci.muni.cz

**Abstract:** Predatory venom is an adaptive trait that has evolved in many different taxa. It is a mixture of various compounds, such as small molecules, peptides and proteins that allow predators to immobilize different prey species. Recently it was found that the composition of spider venom is adapted to their prey. To test the hypothesis that the venom of species that catch ants is more potent against ants than the venom of species that avoid ants, we investigated the efficacy of euryphagous spider venoms. We used 20 species of spiders, each belonging to a different family: *Ariadna* sp. (Segestriidae), *Argiope bruennichi* (Araneidae), *Callobius claustrarius* (Amaurobiidae), *Cheiracanthium* sp. (Cheiracanthiidae), *Dysdera crocata* (Dysderidae), *Eresus kollari* (Eresidae), *Eusparassus duffouri* (Sparassidae), *Filistata insidiatrix* (Filistatidae), *Gnaphosa lucifuga* (Gnaphosidae), *Hogna radiata* (Lycosidae), *Meta* sp. (Tetragnathidae), *Misumena vatia* (Thomisidae), *Palpimanus gibbulus* (Palpimanidae), *Pisaura mirabilis* (Pisauridae), *Philaeus chrysops* (Salticidae), *Selamia reticulata* (Zodariidae), *Steatoda* sp. (Theridiidae), *Tegenaria domestica* (Agelenidae), *Uroctea durandi* (Oecobiidae) and *Zoropsis spinimana* (Zoropsidae). We extracted the crude venom from these spiders by electrical stimulation and injected it at various concentrations into ants and flies. We recorded the mortality after 1, 3 and 24 h and estimated the affected dose (AD50) and the lethal dose (LD50). Comparison of AD50 (1 h) and LD50 (24 h) values across spider species reveals an evolutionary optimisation of venom functionality. The consistently lower LD50 values for flies compared to ants across most species suggest evolutionary adaptations that may reflect the relative ease of subduing dipteran prey versus defended ants. There was a negative correlation between ant acceptance and venom potency. Other factors may influence ant predation strategies, such as specific hunting tactics, behavioural adaptations, or the ability to circumvent ant defences. These data shed new light on the complex evolutionary and ecological factors shaping venom composition in spiders.

**Keywords:** ant-eating spider; euryphagous spider; spider venom; venom efficacy

### 4.14. Otorhinolaryngological Sequelae of Severe Scorpion Envenomation in Children: Case Reports

Tássia Eduarda Ferraz De Almeida ^1^, Mariana Maldonado Loch ^1^, Carolina Sponchiado Miura ^1^, Eduardo Tanaka Massuda ^1^, Fabiana Cardoso Pereira Valera ^1^, Eliane Candiani Arantes ^2^ and Andréa Arantes Braga ^1^,*

^1^  Department of Ophthalmology, Otorhinolaryngology and Head and Neck Surgery, Ribeirão Preto Medical School, University of São Paulo, Ribeirão Preto, SP, Brazil
^2^  Department of Biomolecular Sciences, School of Pharmaceutical Sciences of Ribeirão Preto, University of São Paulo, Ribeirão Preto, SP, Brazil
*  Correspondence: andrea_a_braga@yahoo.com.br

**Abstract:** Severe scorpion envenomation in children, particularly by *Tityus serrulatus*, the most prevalent species in Brazil, remains a major public health challenge. Although acute manifestations are well documented, long-term sequelae such as laryngeal complications and cognitive deficits are poorly characterized. Over the past decade, four paediatric patients at the Clinical Hospital of Ribeirão Preto developed severe subglottic stenosis (SGS) following *T. serrulatus* envenomation. Two were available for detailed follow-up: both required tracheostomy and multiple airway dilations despite orotracheal intubation (OTI) for less than five days. One child exhibited cognitive and learning difficulties and the other developed an occipital pressure ulcer. These cases show that severe scorpion envenomation can lead to persistent laryngeal and neurocognitive sequelae even after brief intubation. While prolonged OTI is a recognized risk factor for SGS, our findings suggest that venom-induced autonomic storm and agitation may potentiate airway injury. Intense venom-driven vasoconstriction may further synergize with airway management factors to precipitate SGS. Early recognition, meticulous airway care and preventive strategies are therefore essential to reduce long-term complications.

**Keywords:** cognitive impairment; learning difficulty; long-term sequelae; scorpion envenomation; subglottic stenosis; *Tityus serrulatus*

## 5. Poster Presentations

Eighteen confirmed and young toxinologists were allowed to present their work as posters—the abstracts of 16 of them are presented below (when more than one author, the underlined name is that of the presenter). Furthermore, six posters were presented as 3-min flash talks—the four of them that are published below are indicated by a #.

### 5.1. Exploring the Dual Role of Cerastes cerastes L-Amino Acid Oxidase: Toxicity and Therapeutic Potential

Zaineb Abdelkafi-Koubaa ^1,2,3,^*, Maram Morjen ^3^, Jed Jebali ^3^, Ines Elbini-Dhouib ^3^, Raoudha Doghri ^1,2^, Karima Mrad ^1,2^, Najet Srairi-Abid ^3^ and Naziha Marrakchi ^3,4^

^1^  Laboratory of Precision Medicine, Personalized Medicine and Oncology Investigation (LR21SP01), Salah Azaiez Institute, University Tunis El Manar, Tunis, Tunisia
^2^  Pathology Department A, Salah Azaiez Institute, Tunis, Tunisia
^3^  Pasteur Institute of Tunis, LR20IPT01, Laboratory of Biomolecules, Venoms and Theranostic Applications, 1002 Tunis, Tunisia; University of Tunis El Manar, 1068 Tunis, Tunisia
^4^  Faculty of Medicine of Tunis, 1068 Tunis, Tunisia
*  Correspondence: abdelkafi_zaineb@yahoo.fr

**Abstract:** L-amino acid oxidase (CC-LAAO) was isolated and thoroughly characterized from *Cerastes cerastes* snake venom. This homodimeric, glycosylated flavoprotein (~58 kDa per subunit, of 498 amino acid residues) exhibited optimal enzymatic activity at pH 7.8 and 50 °C, demonstrating remarkable thermal stability compared to other snake venom LAAOs. Functionally, CC-LAAO exerts a broad spectrum of pharmacological activities primarily mediated by hydrogen peroxide (H_2_O_2_) generated during its enzymatic activity. The enzyme selectively induced cytotoxicity in cancer cells, promoted platelet aggregation, and displayed potent antimicrobial activity against both Gram-positive and Gram-negative bacteria. Binding analysis indicated that CC-LAAO does not directly interact with membrane phospholipids but rather associates with cell surfaces, leading to localized oxidative stress. In vivo acute exposure to low concentrations (1 and 2.5 µg/mL) did not significantly alter key biochemical parameters in mice, including AST, ALT, LDH, and creatinine, suggesting preserved hepatic and renal functions. Histopathological examination confirmed minimal tissue alterations at these doses, supporting a favorable safety profile. Conversely, higher concentrations induced dose-dependent inflammation and necrosis in multiple organs, characterized by immune cell infiltration and structural tissue disruption, thus defining a toxicity threshold relevant to therapeutic development. Importantly, in vitro assays demonstrated that CC-LAAO triggers apoptosis in human glioblastoma U87 cells predominantly through H_2_O_2_-mediated oxidative stress, highlighting its potential as an anti-cancer candidate. Overall, the study delineates the dual nature of CC-LAAO, balancing its cytotoxic and therapeutic properties. The detailed biochemical, histopathological and functional characterization presented here provides a robust foundation for the further development of this venom-derived enzyme as a pharmacological tool or lead compound in toxin-based cancer therapeutics.

**Keywords:** cancer; L-amino acid oxidase; toxicity

### 5.2. Anti-Inflammatory Effects of Dexamethasone in Murine Models Envenomed with Amazonian Tityus Scorpion Venoms

Karina Furlani Zoccal ^1,2^, Karla De Castro Figueiredo Bordon ^2^, Mouzarllem Barros Reis ^2^, Jonas Gama Martins ^3^, Rudi Emerson De Lima Procópio ^4^ and Eliane Candiani Arantes ^2,^*

^1^  Centro Universitário Barão de Mauá, School of Medicine, Ribeirão Preto, São Paulo, Brazil
^2^  University of São Paulo, School of Pharmaceutical Sciences of Ribeirão Preto, Ribeirão Preto, São Paulo, Brazil
^3^  National Institute for Amazon Research, Graduated Program in Genetics, Conservation, and Evolutionary Biology, Manaus, Amazonas, Brazil
^4^  University of the State of Amazonas, Graduated Program in Biotechnology and Natural Resources of Amazon, Manaus, Amazonas, Brazil
*  Correspondence: ecabraga@fcfrp.usp.br

**Abstract:** Scorpion envenomation is a significant public health concern in tropical and subtropical regions, causing intense systemic manifestations and, in severe cases, death. Among clinically relevant species, *Tityus* venoms contain complex toxin repertoires that trigger intense inflammatory responses and critical clinical outcomes. We evaluated if a single dose of dexamethasone, a potent glucocorticoid, could modulate the inflammatory response and clinical severity induced by venoms of different *Tityus* species from the Brazilian Amazon (*T. silvestris*, *T. metuendus*, and *T. obscurus*) and *T. serrulatus*. Mice inoculated with *Tityus* venoms developed a pronounced inflammatory response, evidenced by significantly higher leukocyte and neutrophil counts compared to the control group. Venom-challenged animals also exhibited marked clinical alterations, including lethargy, ocular and nasal secretions, polyuria, and seizures, reflected in elevated clinical scores. Dexamethasone administration 15 min after venom injection significantly attenuated these manifestations. Neutrophil recruitment was significantly reduced, and clinical scores decreased substantially, indicating mitigation of venom-induced systemic severity. In addition, dexamethasone treatment demonstrated a pronounced effect on cytokine modulation. Treated animals exhibited a significant increase in interleukin-10, an anti-inflammatory cytokine known to downregulate pro-inflammatory pathways and limit tissue damage. Additionally, interleukin-6, a central mediator of acute inflammatory responses, was significantly reduced after treatment. This cytokine shift toward an anti-inflammatory profile provides mechanistic support for dexamethasone-mediated protection against venom-induced pathology. Dexamethasone treatment also conferred complete protection against *Tityus* venom-induced lethality. All treated animals survived the six-hour monitoring period, regardless of the venom source. In contrast, survival was drastically reduced in vehicle-treated groups. Mice inoculated with *T. serrulatus* venom died within 2 h post-injection, whereas those challenged with *T. silvestris* exhibited mortality within 6 h. Mortality rates of 60% and 40% were recorded for *T. metuendus* and *T. obscurus*, respectively, under untreated conditions. These results demonstrate that dexamethasone can mitigate venom-induced inflammation and clinical outcomes, preventing mortality in the murine model. This study underscores the therapeutic potential of anti-inflammatory strategies as adjunctive interventions in scorpionism, particularly considering the rapid onset and high lethality associated with certain *Tityus* species.

**Support:** FAPESP (2021/11936-3, 2024/16842-5, 2025/01816-1), CNPq (309399/2021-1, 169589/ 2023-4).

**Keywords:** dexamethasone; envenomation; IL-10; IL-6; inflammation; *Tityus*

### 5.3. Mass Spectrometry-Based Imaging and Molecular Profiling of Snake Venom Effects on Murine Tissues ^#^

Quentin Bastiaens ^1,^*, Axel De Monts De Savasse ^1^, Virginie Bertrand ^1^ and Loïc Quinton ^1^

^1^  Mass Spectrometry Laboratory, MolSys Research Unit, Quartier Agora, University of Liège, allée du Six Août 11, B-4000 Liège, Belgium
*  Correspondence: q.bastiaens@uliege.be

**Abstract:** Snake envenomation is classified as a Neglected Tropical Disease and is responsible for approximately 100,000 fatalities worldwide each year. In addition to its high mortality, snake venom is a complex biochemical mixture composed predominantly of proteins and peptides collectively referred to as toxins. These toxins display a broad spectrum of biological activities, including neurotoxic, hemotoxic, and cytotoxic effects, which can severely damage various tissues and organ systems. This thesis aims to develop and optimize a mass spectrometry-based workflow to investigate and visualize the molecular effects of snake venom on murine tissues. The overarching objective is to elucidate the biochemical and spatial alterations induced by venom exposure in tissues commonly affected during envenomation, such as skeletal muscle, liver, heart, and brain. Experimental models will involve the controlled application of snake venom to these tissues, followed by analysis to characterize the resulting molecular perturbations. Moreover, to assess potential therapeutic interventions, selected venom inhibitors and commercially available antivenoms will be co-incubated with venom-treated tissues to evaluate their efficacy in neutralizing toxin activity and mitigating tissue degradation. The project comprises two complementary components: mass spectrometry imaging (MSI) and liquid chromatography coupled with tandem mass spectrometry (LC-MS/MS). The imaging component will employ matrix-assisted laser desorption/ionization MSI (MALDI-MSI), enabling both the acquisition of high-resolution mass spectra and the spatial localization of ionized species across tissue sections. This approach provides critical insight into the distribution of venom-derived and host-response molecules within the affected tissues. The second component involves LC-MS/MS, which will be used for detailed molecular profiling and identification. This analytical strategy will facilitate the detection and structural characterization of specific toxins, degradation products, and host-derived biomolecules implicated in venom-induced pathophysiology. Together, these methodologies aim to provide a comprehensive understanding of snake venom action at the molecular level as well as evaluate the mechanistic basis of antivenom and inhibitor efficacy.

**Keywords:** mass spectrometry; mass spectrometry imaging; mice tissue; snake PLA2; toxin; venom

### 5.4. Characterisation of New Toxins in the Marine Cyanobacteria Trichodesmium erythraeum

Léa Maupetit ^1^, Theo-Bob Muller ^2^ and Isabelle Catherine Biegala ^1,^*

^1^  Aix-Marseille Université, Avignon Université, CNRS, IRD, IMBE, 13007 Marseille, France
^2^  Aix-Marseille Université, INSERM, SSA, MCT, 13385 Marseille, France
*  Correspondence: isabelle.biegala@imbe.fr

**Abstract:** The well-known cyanobacteria *Trichodesmium erythraeum* are associated with public health issues in the Pacific. Although the hepathotoxins microcystins are well spread in freshwater cyanobacteria, few studies have mentioned their presence in *T. erythraeum* marine blooms. In this study, 51 leucine-arginine (MC-LR) variants were identified by LC-MS/MS and MALDI-ToF in *T. erythraeum* blooms. They were obtained using an original approach that involved reconstituting molecules from ions fragmented in the ionization source. Among them, three structures were already known from freshwater, 31 were fully characterized, and 20 structures were only partially characterized. Their structures were distinguished by propylated D-glutamate, glycine, and alanine in key microcystin positions. Some of the structures identified suggested a stronger toxicity than that of MC-LR, as a third of the marine variants were more lipophilic, and 42% had variables that could increase their toxicity. According to the global oceanic distribution of *T. erythraeum*, these results underline the need for health monitoring in the marine environment, similar to the one applied in freshwater by the WHO.

**Keywords:** cyanobacteria; LC-MS/MS; MALDI-ToF marine; microcystin; *Trichodesmium*

### 5.5. Immunomodulatory Activities of the MYRTXA1-Tb1a Peptide from the Venom of the Ant Tetramorium bicarinatum

Stéphanie Long, Bilal Arrahouti, Elsa Bonnafé and Arnaud Billet *

Equipe BTSB-EA, Université de Toulouse, Institut National Universitaire Jean-François Champollion, Place de Verdun, 81012, Albi, France

*  Correspondence: arnaud.billet@univ-jfc.fr

**Abstract:** Ants represent a phylogenetic group whose venoms have long remained little studied. It has previously been demonstrated that their venoms exhibit high diversity in their composition (alkaloids, proteins, peptides). Our research group previously deciphered the venoms of several ant species and showed they were mainly composed of peptides. Venom peptides are used for defense and predation and, as such, have cytotoxic, paralyzing or nociceptive effects. However, we recently found that several putative GATA transcription factor binding sites are localized in many promoters of venom peptide genes. Since GATA factors play roles in insect immunity, we wondered whether some venom peptide genes might have been recruited from those involved in immune functions. In this study we explored the role of venom peptide in ant immunity. Preliminary data showed that venom peptide genes were expressed in the abdomen, outside the gland venom. As the two main immune centers in insect are the fat body and the hemocytes, we evaluated their expression in these two tissues and showed that venom peptide genes were selectively expressed in hemocytes and mature peptides secreted in hemolymph. Indeed, we detected several venom peptides in hemolymph with MRTXA1-Tb1a as one of the most abundant peptides. While MYRTXA1-Tb1a has already shown immunomodulatory activities on mammalian cells through GPCR activation and calcium mobilization [1,2], we decided to assess its immunomodulatory effects on S2 cells, a *Drosophila melanogaster* hemocyte-derived cell line. We evaluated its ability to modulate immune pathways by RNA-sequencing and compared the effects to those of exposure to PGN and 20-hydroxyecdysone, two potent inducers of immunity in *Drosophila*. Exposure to MYRTXA1-Tb1a induced a strong underexpression of some Heat Shock Proteins (HSPs) in S2 cells, which are known to play key roles in regulating stress-related immune responses in insects. In addition to these inhibitory effects, MYRTXA1-Tb1a induced phagocytosis in S2 cells without any calcium mobilization. Therefore, MYRTXA1-Tb1a appears to have endogenous immunomodulatory effects, indicating a close link between innate immunity and venomous function in ants. However, the exact mechanisms of immunomodulatory activity and the associated molecular pathways remain to be determined.

**Keywords:** ant venom; immunity; peptide

**References:**

1. Benmoussa, K.; Authier, H.; Prat, M.; AlaEddine, M.; Lefèvre, L.; Rahabi, M.C.; Bernad, J.; Aubouy, A.; Bonnafé, E.; Leprince, J.; et al. P17, an original host defense peptide from ant venom, promotes antifungal activities of macrophages through the induction of C-type lectin receptors dependent on LTB4-mediated PPARγ activation. *Front. Immunol.* **2017**, *8*, 1650.
2. Duraisamy, K.; Singh, K.; Kumar, M.; Lefranc, B.; Bonnafé, E.; Treilhou, M.; Leprince, J.; Chow, B.K.C. P17 induces chemotaxis and differentiation of monocytes via MRGPRX2-mediated mast cell-line activation. *J. Allergy Clin. Immunol.* **2022**, *149*, 275–291.

### 5.6. Probing Folding Kinetics and Stability of Therapeutic Toxins Through Site-Specific Cysteine-to-Selenocysteine Substitution ^#^

Chloé Cayrou ^1^, Nicolas Gilles ^1^, Pascal Kessler ^1^, Philippe Cuniasse ^2^, Bernard Maillere ^1^, Denis Servent ^1,^* and Nicolo Tonali ^1,^*

^1^  Université Paris Saclay, CEA, Département Médicaments et Technologies pour la Santé (DMTS), SIMoS, Gif-sur-Yvette, France
^2^  Université Paris Saclay, CEA, CNRS, Institute for Integrative Biology of the Cell (I2BC), Gif-sur-Yvette, France
*  Correspondence: denis.servent@cea.fr (D.S.); nicolo.tonali@cea.fr (N.T.)

**Abstract:** Many bioactive toxins depend on intricate disulfide networks to acquire and maintain their biologically active conformations. However, these disulfide bridges are highly susceptible to cleavage in reducing environments, leading to structural destabilization and loss of activity. This intrinsic fragility limits the therapeutic potential of cysteine-rich toxins. Substituting selected cysteine residues with selenocysteine (Sec) offers an elegant strategy to overcome these limitations. The resulting diselenide (Se–Se) bridges are almost isostructural to native disulfides but display a lower pKa and a markedly more negative redox potential, enabling faster oxidative formation and greater resistance to reduction [1]. Pioneering studies on diselenide-substituted peptides (including insulin [2], apamin [3], and several conotoxin analogues [4]) have demonstrated that selenocysteine incorporation can (i) direct oxidative folding toward the native connectivity, (ii) preserve overall structure and biological function, and (iii) enhance stability in reducing environments. In this work, we explored the potential of selenocysteine substitution in two snake venom toxins: mambalgin-1 (three-finger toxin) and mambaquaretin-1 (Kunitz-type toxin). Using optimized solid-phase peptide synthesis, Sec residues were selectively introduced at key cysteine positions to generate selenium-containing analogues. These Se-toxins were characterized through structural, biochemical, and preliminary functional analyses to evaluate how sulfur-to-selenium substitution influences oxidative folding, redox stability, and activity. Moreover, comparative analyses with native forms will help delineate the specific contribution of selenium chemistry to conformational resilience and activity retention. Altogether, this work provides new molecular insights into how selenocysteine can strengthen the structural robustness of disulfide-rich scaffolds and highlights Sec incorporation as a promising and generalizable approach for designing redox-stable, bioactive venom peptides and toxins.

**Keywords:** Kunitz-type toxin; selenocysteine; three-finger toxin

**References:**

1. Cheng, X.; Wu, C. Directing the oxidative folding of disulfide-rich peptides for enhanced engineering and applications. *Chem. Sci.* **2025**, *16*, 19012.
2. Weil-Ktorza, O.; Rege, N.; Lansky, S.; Shalev, D.E.; Shoham, G.; Weiss, M.A.; Metanis, N. Substitution of an internal disulfide bridge with a diselenide enhances both foldability and stability of human insulin. *Chem. Eur. J.* **2019**, *25*, 8513–8521.
3. Wang, X.; Wu, J.; Liu, X.; Tang, K.; Cheng, L.; Li, J.; Tang, Y.; Song, X.; Wang, X.; Li, C. Engineered liposomes targeting the gut–CNS axis for comprehensive therapy of spinal cord injury. *J. Control. Release* **2021**, *331*, 390.
4. Kennedy, A.C.; Belgi, A.; Husselbee, B.W.; Spanswick, D.; Norton, R.S.; Robinson, A.J. α-Conotoxin peptidomimetics: Probing the minimal binding motif for effective analgesia. *Toxins* **2020**, *12*, 505.

### 5.7. Development of an Analytical Framework to Evaluate Antivenom Efficacy Through Magnetic Beads and Biolayer Interferometry ^#^

Thomas Crasset ^1^, Germain Behonsé ^1^, Damien Redureau ^1^, Fernanda Gobbi Amorim ^1^, Nicholas Casewell ^2^, Stefanie Menzies ^3^, Christiane Berger-Schaffitzel ^4^, Marylène Vandevenne ^5^ and Loïc Quinton ^1,^*

^1^  Laboratory of Mass Spectrometry, MolSys Research Unit, University of Liège, Liège, Belgium
^2^  Centre for Snakebite Research and Interventions, Liverpool School of Tropical Medicine, Pembroke Place, Liverpool, UK
^3^  Department of Biomedical and Life Sciences, Lancaster University, Lancaster, UK.
^4^  School of Biochemistry, University of Bristol, Bristol, UK
^5^  Laboratory of Enzymology and Protein Folding, Centre for Protein Engineering, InBioS, University of Liège, Liège, Belgium
*  Correspondence: thomas.crasset@uliege.be

**Abstract:** Snake envenomation remains a major public health concern in Africa, the Middle East, Asia, and subtropical regions, particularly affecting rural communities. Each year, between 80,000 and 138,000 people die from snakebite envenomation, and three times as many suffer long-term disabilities despite treatment. For this reason, the World Health Organization has classified snakebite envenomation as a Category A neglected tropical disease. Although treatments are available, antivenoms remain the only therapeutic option currently on the market and suffer from several limitations. These purified sera contain not only toxin-specific IgGs but also non-specific IgGs and, sometimes, additional serum proteins, which can sometimes trigger adverse reactions. Moreover, antivenoms are thermally unstable and difficult to store in regions where temperatures can be extreme. To address these issues, the European ADDovenom project (2021–2025, EC-FET-Open) aims to develop a new generation of antivenoms based on ADDomers: cost-efficient, thermally stable, megadalton-scale virus-like particles with 60 high-affinity binding sites, offering a promising alternative. In this study, we developed a new methodology for toxin identification, quantification, and affinity measurement using a fast and fully automatable workflow. Tosylactivated or Protein G-coupled magnetic beads were coupled to EchiTab G, a monospecific antivenom targeting *Echis ocellatus* toxins, and tested against *E. romani*, a related species described by Trape et al. in 2018 [1]. Eluates and supernatants were analyzed by LC-MS/MS after tryptic digestion to identify and quantify toxins immuno-recognized by EchiTab G. In parallel, biolayer interferometry (BLI) was used to measure the apparent dissociation constants of whole antivenoms against whole venoms, enabling comparison with alternative treatments such as nanobodies, monoclonal antibodies, or ADDobody-based structures. These fully automatable methods require minimal sample amounts and could be implemented in routine quality control workflows to improve antivenom efficacy assessment.

**Keywords:** antivenomics; liquid chromatography; mass spectrometry; method development; snake venom

**References:**

1. Trape, J.-F. Partition d’*Echis ocellatus* Stemmler, 1970 (Squamata, Viperidae), avec la description d’une espèce nouvelle. *Bull. Société Herpétologique Fr.* **2018**, *167*, 13–34.

### 5.8. Predatory Versus Defensive Venom Uses in Textilia bullatus

Zahrmina Ratibou ^2^, Valentin Dutertre ^2^, Nicolas Inguimbert ^2^ and Sébastien Dutertre ^1,^*

^1^  IBMM, Université de Montpellier CNRS, ENSCM, 34095 Montpellier, France
^2^  CRIOBE, USR 3278-EPHE-CNRS-UPVD, Université de Perpignan via Domitia, 58 Avenue Paul Alduy, 66860 Perpignan, France
*  Correspondence: sebastien.dutertre@umontpellier.fr

**Abstract:** In animals, venom is a sophisticated biological weapon used for both predation and defense. Composed of complex cocktails of peptides, proteins, and enzymes, venoms target specific physiological pathways, such as nervous or cardiovascular systems. Many species—including snakes, spiders, scorpions, insects, and cone snails—have developed venom glands and specialized injection mechanisms (fangs, stingers, harpoons) that allow them to quickly immobilize prey or deter predators. In a predatory context, venom often acts by rapidly paralyzing or killing prey, making it easier to capture and digest. Conversely, in defensive situations, venom typically causes immediate and intense pain, inflammation, or sensory disruption in the aggressor, prompting it to retreat. The remarkable ability of some cone snails to produce distinct venoms in response to predatory or defensive stimuli was unexpected, implying a specific and directed evolution for both purposes. To date, the mechanism underlying toxin selection remains unknown, and more studies are needed to help understand it. Here, I will provide the first insights into the predatory and defensive venoms of a piscivorous species of cone snail from the *Textilia* clade. Quite remarkably, the composition of both venoms shows almost no overlap, and these findings further highlight the dynamic interplay between ecology, behaviour, and evolution.

**Keywords:** cone snail; conotoxin; defence; predation

### 5.9. Endothelial Barrier Disruption by CdtVEGF: Insights in Human Umbilical Vein Endothelial Cells

Isabela Ferreira ^1,2,3^, James Hallwood ^3^, David Bates ^3^, Andrew Benest ^2^ and Eliane Candiani Arantes ^1,^*

^1^  School of Pharmaceutical Sciences of Ribeirão Preto, University of São Paulo, Ribeirão Preto, São Paulo, Brazil
^2^  Endothelial Quiescence Group, Centre for Cancer Sciences, Biodiscovery Institute, School of Medicine, University of Nottingham, Nottingham, United Kingdom
^3^  Tumour and Vascular Biologies Laboratory, Biodiscovery Institute, School of Medicine, University Nottingham, Nottingham, United Kingdom
*  Correspondence: ecabraga@fcfrp.usp.br

**Abstract:** Snake venoms constitute complex mixtures enriched with bioactive molecules, predominantly proteins exhibiting potent pharmacological activities. Nevertheless, the isolation of native proteins from these venoms often results in low recovery yields. For example, in rattlesnake venom (*Crotalus durissus terrificus*, Cdt), a homodimeric vascular endothelial growth factor (VEGF) with molecular mass of 25 kDa was recovered as 2% of the total venom. Since VEGFs have the ability to promote angiogenesis and increase vascular permeability through direct interaction with VEGF receptors (VEGFRs), in this study we analysed native CdtVEGF, isolated from Cdt venom, regarding its capacity to promote vascular permeability on human umbilical vein endothelial cells (HUVECs). First, HUVECs cells were seeded onto coverslips in a 24-well plate (2 × 10^5^ cells/well) previously coated with 1% gelatin. After 48 h, cells were stimulated with CdtVEGF and VEGFA165 (1 nM) for 5, 10, 15, 30 and 60 min. After that, cells were fixed and stained using anti-VE-Cadherin antibody (5 µg/mL) and DAPI (1:500) and analysed on a confocal microscope. Our results demonstrated some slightly change in cell-cell junction, with a zig-zag pattern on the cell membrane after being treated after 60 min with both VEGFs. So, to investigate more of the permeability activity of CdtVEGF we performed an Electric Cell-substrate Impedance Sensing (ECIS) assay. For this one, HUVECs cells were seeded (5 × 10^5^ cells/well) into E-chips previously coated with 1% gelatin (8W1E PET, ECIS^®^ Z-Theta) and after 24 h, cells were stimulated with CdtVEGF, VEGFA165 (1 nM and 2 nM) and TNF-alpha (10 ng/mL) and the impedance was recorded by the ECIS software for 72 h. Our results showed that VEGFA165 and CdtVEGF were capable of inducing changes at the barrier properties of endothelial cell junctions. Our findings help shed light on the role of snake venom VEGFs in modulating the endothelial cell barrier, contributing to an increased vascular permeability.

**Keywords:** permeability; snake venom; vascular endothelial growth factor

### 5.10. Itch- and Pain-Related Behaviors Induced by Intradermal Ciguatoxin in Mice

Raphaële Le Garrec ^1,^*, Taylor Follansbee ^2^, Mirela Iodi Carstens ^2^, Laurent Misery ^1^ and Earl Carstens ^2^

^1^  Université de Brest, Laboratoire Interactions Épithéliums-Neurones (LIEN), F-29200 Brest, France
^2^  Department of Neurobiology, Physiology and Behavior, University of California, Davis, CA, United States
*  Correspondence: rlegarrec@univ-brest.fr

**Abstract:** Ciguatoxins (CTXs) are the neurotoxins derived from marine microalgae responsible for ciguatera poisoning. The most frequent symptoms are sensory neuropathic disorders, including cold allodynia and a persistent itch. Spontaneous pain (e.g., in muscles and joints) and persistent fatigue are also commonly described. To characterize the spontaneous behaviors elicited by a range of nanomolar doses of pure CTX injected intradermally in mice, we used the mouse cheek test, which distinguishes between itch- and pain-related behaviors. Video recordings of behavioral responses were analyzed to count the number of behaviors reflecting itch and pain. In addition, the other evoked behaviors were noted and/or their durations were measured. Our results show for the first time that itch is the critical effect of CTX in this mouse model, i.e., the first measurable effect to occur in response to increasing doses. Pain-related behaviors occurred at a 100-fold higher dose. In addition, mice exhibited some dose-dependent unexpected behaviors, namely scratching bouts directed toward body areas other than the injected hemiface, and increased durations of total inactivity. These data suggest that CTX applied to the mouse cheek test is a singular tool for studying some major symptoms of ciguatera, especially itch.

**Support:** This project has received funding from the European Union’s Horizon 2020 research and innovation programme under the Marie Sklodowska-Curie grant agreement No 101026260.

**Keywords:** behavior; ciguatoxin; neuropathy

### 5.11. Toxin Hunter: A Next-Gen Biosensor for the Identification and Quantification of Toxins ^#^

Tom Miclot *

CNRS and Université Paris Cité, ITODYS UMR7086, Paris, France

*  Correspondence: tom.miclot@u-paris.fr

**Abstract:** The detection of toxic substances in biological and environmental samples is a crucial challenge in public health, food safety, and clinical diagnostics, requiring methods that are rapid, sensitive, selective, and suitable for real-world applications. Biosensors have emerged as powerful tools for toxin detection by combining a biological recognition element with a physicochemical transducer to generate a measurable signal upon target binding. One promising strategy involves the use of synthetic DNA aptamers. A notable example is the detection of Ochratoxin A (OTA), a mycotoxin commonly found in contaminated food products. In previous studies, OTA has been successfully detected using a DNA aptamer which forms a stable G-quadruplex (G4) structure that selectively recognizes and binds the toxin. Structural studies by molecular dynamics simulations have shown that upon binding, OTA reduced conformational flexibility, reflecting the formation of a rigid and stable complex. When integrated into functionalized surface of a biosensor the OTA–G4 binding event induces measurable changes in electrical current, highlighted by a distribution of ion density in simulations. The use of G-quadruplex-forming aptamers offers several advantages, including ease of synthesis and the possibility of functionalization for surface immobilization. Moreover, these biosensors can be engineered to minimize interference from complex sample matrices, enhancing selectivity and reliability. By leveraging the molecular recognition capabilities of DNA aptamers and translating binding events into quantifiable outputs, biosensor technologies offer a viable alternative to conventional analytical methods like chromatography or immunoassays, which often require expensive equipment, lengthy protocols, and trained personnel. The development of aptamer-based biosensors thus represents a significant advancement toward point-of-care, real-time monitoring of toxins, with broad implications for food quality control.

**Keywords:** biosensor; detection; toxin

### 5.12. Altered Elimination of Polyinnervation in Orbicularis Oculi Muscles of Essential Blepharospasm Patients with Poor Response to Botulinum Toxin Type-A

Brigitte Girard ^1,2^, Aurélie Couesnon ^3^, Emmanuelle Girard ^4^ and Jordi Molgó ^3,5,^*

^1^  Service d’Ophtalmologie—Hôpital Tenon, Sorbonne Université, UPMC, France
^2^  Hôpital Privé Armand Brillard, Nogent sur Marne, France
^3^  Institut des Neurosciences Paris-Saclay, UMR 9197, CNRS/Université Paris-Sud, Gif-sur-Yvette, France
^4^  Université Lyon 1, Physiopathology & Genetic of Neuron and Muscle, CNRS UMR5261, INSERM U1315, Lyon, France
^5^  Université Paris-Saclay, CEA, Département Médicaments et Technologies pour la Sante (DMTS), Service d’Ingénierie Moléculaire pour la Sante (SIMoS), EMR CNRS 9004, Gif-sur-Yvette, France
*  Correspondence: jordi.molgo@cea.fr

**Abstract:** Essential blepharospasm (EB) is a focal cranial dystonia characterized by involuntary, bilateral, and intermittent or sustained eyelid closures due to hyperactivity of the orbicularis oculi muscles. Symptom severity ranges from increased blinking to functional blindness. Although botulinum neurotoxin type-A (BoNT/A) injections remain the first-line therapy, a subset of patients (<4%) exhibit reduced or unsustained responses to repeated treatment, and the underlying mechanisms remain poorly understood. The objective of this work was to investigate morphological alterations of the neuromuscular junction (NMJ) in orbicularis oculi muscles from EB patients showing poor response to BoNT/A compared with BoNT/A-naïve controls. A blinded morphological analysis was conducted on orbicularis oculi muscle specimens obtained during upper myectomy from BoNT/A-treated EB patients and from control individuals undergoing blepharoplasty. Treated patients had received either abobotulinumtoxinA (Dysport^®^, Ipsen Ltd., London, UK) or incobotulinumtoxinA (Xeomin^®^, Merz Pharmaceuticals GmbH, Frankfurt/Main, Germany). NMJ architecture, motor axon innervation patterns, and synaptic remodeling markers were assessed through immunohistochemical techniques and confocal laser scanning microscopy. NMJs from BoNT/A-treated patients exhibited polyneuronal innervation, with one to six motor axons per junction, irrespective of the toxin formulation used. In contrast, 99.47% of NMJs from BoNT/A-naive control samples were monoinnervated, as expected in mature skeletal muscles. The persistence of polyinnervation following repeated BoNT/A exposure suggests impaired synapse elimination, possibly due to altered signaling or dysfunction of perisynaptic Schwann cells. Evidence of synapse elimination, identified by retraction bulbs and partial withdrawal of motor terminals, was found in only 14.28% of poorly responsive patient samples. Notably, postsynaptic nicotinic acetylcholine receptor (nAChR) withdrawal occurred in the presence of intact retraction bulbs, implying that receptor loss may precede nerve terminal retraction. Our findings indicate that repeated BoNT/A exposure disrupts normal synaptic remodeling in the orbicularis oculi muscle, resulting in persistent polyinnervation at mature NMJs. These alterations likely reflect a failure of perisynaptic Schwann cells to coordinate synapse elimination and may represent a cellular mechanism contributing to the diminished therapeutic response observed in a subset of EB patients.

**Keywords:** blepharospasm; botulinum type-A neurotoxin; synapse elimination

### 5.13. Effect of Botulinum Toxin Bont/A1, Injected into the Detrusor, on Urodynamic Parameters in Conscious Rats

Christine Rasetti-Escargueil *, Joïlita Bonnet, Sekou Diarra and Stefano Palea

HUMANA Biosciences, Prologue Biotech, 516 rue Pierre et Marie Curie, Labège, France

*  Correspondence: christine.escargueil@humana-biosciences.com

**Abstract:** Introduction—Onabotulinum toxin A (BoNT/A) was approved for the treatment of Urgency Urinary Incontinence (UUI) by the FDA in 2013 whereas Abobotulinum toxin A received a positive opinion in Europe for the management of neurogenic detrusor overactivity in adults. The aim of this study was to set up an experimental model to study the effects of botulinum toxins type A on the urinary bladder function in conscious rats.

Methods—BoNT/A1 (*n* = 6; complex form) or its gelatin-phosphate vehicle (*n* = 7) was injected into the urinary bladder of young adult male rats (5 U/rat, administered in 5 sites) under anesthesia. Six days later, rats were placed into urination cages (Shinfactory, Fukuoka, Japan) and urodynamic parameters recorded continuously for 24 h. The experiment was performed in a dedicated room of the animal house, with a Light-Dark cycle of 12 h. Voiding Frequency (VF), Urine Volume per Voiding (UVV), Micturition Duration (MD) as well as Urine Production (UP), Water intake (WI) and Food Intake (FI) were expressed as values recorded in Light and Dark Periods of 12 h, as well as values recorded for 24 h.

Results—One week after BoNT/A injections, a statistically significant decrease of VF in both Dark and Light periods was noticed in the BoNT/A treated rats, with respect to vehicle-treated rats. Interestingly, the toxin increased the UVV in Dark Periods only, suggesting that the positive effect on bladder capacity occurred mainly during the rat’s active period of the circadian clock. This conclusion is confirmed by the significant increase in MD in rats treated with BoNT/A, on Dark Periods only. In addition, we noticed a significant decrease in WI and FI following treatment with BoNT/A, probably explaining the marked body weight loss in toxin-treated rats. In preliminary experiments (*n* = 4) the urodynamic parameters in urination cages came back to normal values 14 days after BoNT/A injection. Moreover, 14 days post-injection, FI in BoNT/A treated rats was similar to values in vehicle-treated rats.

Conclusion—We have validated a new experimental model in conscious rats to study the effects of intra-detrusor injections of BoNT/A on the urodynamic parameters. The effect of BoNT/A at 5U is detectable 7 days following intra-detrusor injection, but not at day 14 post-injection. Therefore, our non-invasive experimental model is suitable to compare the effects of new recombinant botulinum toxins, more suitable than native toxins for the pharmacological treatment of lower urinary tract pathologies.

**Keywords:** botulinum toxin; conscious rat; neurogenic detrusor overactivity

### 5.14. Exploring Middle Eastern Animal Venoms as Natural Sources of Anti-Senescence Compounds

Mona El Samarji ^1^, Elissa Alam ^1^, Ziad Fajloun ^2,3^ and Mohamad Rima ^1,^*

^1^  Department of Biological Sciences, Lebanese American University, Byblos P.O. Box 36, Lebanon
^2^  Laboratory of Applied Biotechnology (LBA3B), Azm Center for Research in Biotechnology and Its Applications, EDST, Lebanese University, 1300 Tripoli, Lebanon
^3^  Faculty of Sciences 3, Michel Slayman Tripoli Campus, Lebanese University, Ras Maska 1352, Lebanon
*  Correspondence: mohamad.rima@lau.edu.lb

**Abstract:** Senopathies, or age-related diseases, are attracting growing interest due to their strong association with cellular senescence and chronic inflammation. While a wide range of treatments is currently available, naturally derived agents, particularly those from animal venoms, remain poorly studied as candidates for anti-senescence therapy. In this study, we investigated the potential activity of Middle Eastern animal venoms in senescence models of MDA-MB-231 and MCF-7 breast cancer cells. After induction and validation of the senescent phenotype, venom cytotoxicity was assessed in both senescent and proliferating cells. Our results demonstrate that the venom exerts a significant cytotoxic effect on both cell populations, with a notably lower EC50 in senescent cells, indicating a preferential senolytic effect. Preliminary compound screening identified the bioactive components responsible for the senolytic effect. Our current focus is to elucidate the mode of cell death induced by the venom and its bioactive constituents in both proliferating and senescent cells. Overall, our study highlights the therapeutic promise of this venom as a natural source of senolytic agents, with potential applications in cancer therapy and the management of age-related diseases.

**Keywords:** animal venom; natural compound; senescence; senolytics

### 5.15. Is Cannabidiol (CBD) Toxic?

Dušan Šuput ^1,2,^*, Klara Bulc Rozman ^1^ and Miran Brvar ^1,3^

^1^  Centre for Clinical Physiology, Faculty of Medicine, University of Ljubljana, Zaloska Cesta 4, 1000 Ljubljana, Slovenia
^2^  Academic Research Centre of the Medical Association of Slovenia, Dunajska 162, 1000 Ljubljana, Slovenia
^3^  Centre for Clinical Toxicology and Pharmacology, University Medical Centre Ljubljana, Zaloška Cesta 7, 1000 Ljubljana, Slovenia
*  Correspondence: dusan.suput@mf.uni-lj.si

**Abstract:** Introduction—The use of cannabidiol (CBD) as a drug or supplemental drug is increasing as there are many reports promoting its beneficial effects. It is considered non-toxic and its possible effects on brain development are largely unknown. Considering the potential use of CBD in children or during pregnancy, we studied its effects on rat neonatal neurons and astrocytes.

Methods—All experiments were performed on perinatal rat cortical neurons and astrocytes exposed to 0.1, 0.5, and 1.5 µM CBD, as such concentrations can be reached in human bodies.

Results—In neurons, 0.1 µM CBD transiently decreased the mitochondrial membrane potential, followed by ATP depletion, and triggered the apoptotic pathway. Astrocytes were more resistant. Identical effects as in neurons were observed only at 0.5 µM CBD concentration. In both cell types, the effects of CBD could be prevented by antagonists of the transient receptor potential vanilloid type 1 (TRPV1) or of the cannabinoid receptor (CB1).

Conclusion—CBD is toxic to neonatal neurons and astrocytes. This should be considered seriously as the blood-brain barrier is not fully functional during the neonatal and early postnatal period.

**Keywords:** astrocyte; cannabidiol; neonatal; neuron; toxic

### 5.16. Novel Disintegrins Isolated from Bothrops jararaca Venom: Partial Characterization and Antitumor Potential

Adelia Cristina De Oliviera Cintra, Gabriel Cezarette, Ciro Pedro G. Pinto, Thiago Abrahão Silva, João José Franco and Suely Vilela Sampaio *

Department of Clinical Analysis, Toxicology and Food Science, School of Pharmaceutical Sciences of Ribeirão Preto, University of São Paulo, SP, Brazil

*  Correspondence: suvilela@usp.br

**Abstract:** Snake venoms are complex mixtures of bioactive molecules with a broad pharmacological spectrum and recognized therapeutic potential. Among these components, disintegrins stand out. They are low molecular weight peptides capable of inhibiting integrins, thereby modulating biological processes such as cell adhesion, migration, angiogenesis, and metastasis. The present study aimed to isolate and characterize novel disintegrins from *Bothrops jararaca* venom, as well as to evaluate their antitumor potential and the mechanisms associated with inhibition of tumorigenesis in vitro. Isolation was performed using two successive stages of reverse-phase liquid chromatography (C18), resulting in highly pure fractions. The isolated disintegrins, named Bjara-I and Bjara-II, were evaluated for their effects on cell migration and proliferation using the HepG2 human hepatocellular carcinoma cell line. Both samples significantly reduced cell migration and proliferation at concentrations ranging from 4 to 16 µg/mL compared to control groups. Interaction with adhesion receptors was investigated through platelet aggregation assays using platelet-rich and platelet-poor plasma, with ADP as the agonist. Bjara-II showed complete inhibition (100%) of ADP-induced platelet aggregation at a concentration of 8 µg/mL, indicating affinity for the αIIbβ3 integrin. In contrast, Bjara-I did not exhibit inhibitory effects, suggesting selectivity for other integrin subtypes possibly related to tumor cell adhesion. Mass spectrometry analysis (MALDI-TOF) revealed intact molecular masses of 7770 Da for Bjara-I and 7364 Da for Bjara-II. These results indicate that *B. jararaca* venom contains new disintegrins in addition to the already known ones (jararacin and jarastatin), with distinct structural and functional properties. The identification of a disintegrin with selective antitumor action and no antiaggregant activity is a relevant finding, opening perspectives for structure-function relationship studies and for the development of new drug candidates with antimetastatic potential.

**Keywords:** cell migration; disintegrin; platelet aggregation

## 6. Conclusions

The RT31 meeting of the SFET was a success, with a highly international, well balanced in terms of gender and professional level, and eclectic audience. As always, the youngest participants (about a quarter of the total audience) were given prominence for the presentations. Compared to the previous RTs, there were two new features: a plenary conference and a gala dinner. Both were very well received by the participants and will be repeated for the next RT32 meeting, which is already being organized—stay tuned!

## 7. Acknowledgments

**Figure 4:**
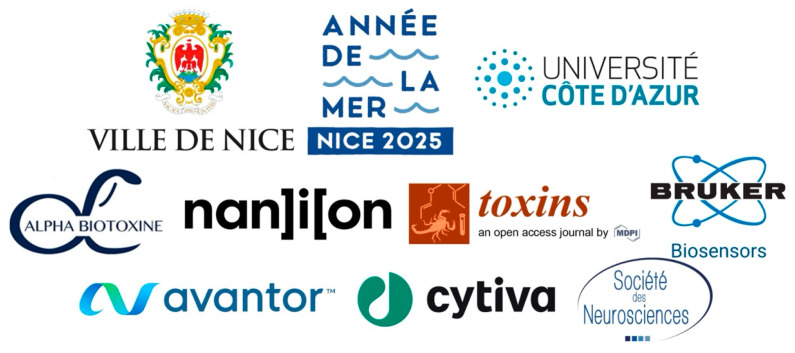
The sponsors (first two lines) and other supporters (third line) of the 31st Meeting on Toxinology (RT31) of the French Society for Toxinology (SFET).

We gratefully acknowledge the commitment and sustained efforts of all our colleagues whose work contributes to the national and international visibility of the SFET. Our sincere appreciation also goes to all those who contributed to the success of this RT31 meeting. We extend our special thanks to our long-standing sponsors and to those who supported the meeting for the first time for their essential and valuable contributions (Figure 4). Finally, we thank the journal *Toxins* (MDPI) for its commitment to publishing this conference report and to the reviewers for their kind and helpful suggestions.

## Data Availability

No new data were created or analyzed in this study.

